# Design, Synthesis, and Biological Evaluation of Pyrazol‐5‐yl‐Phenylacetamide Derivatives as Potential Fungicidal Agents

**DOI:** 10.1002/cbdv.71784

**Published:** 2026-09-27

**Authors:** Guo‐Tai Lin, Wei Wang, Xi‐Zhe Zhang, Li Luo, Gong Xu, Dan Xu

**Affiliations:** ^1^ Shaanxi Key Laboratory of Natural Products and Chemical Biology, College of Chemistry & Pharmacy Northwest A&F University Yangling Shaanxi China; ^2^ Key Laboratory of Plant Protection Resources and Pest Management of Ministry of Education College of Plant Protection Northwest A&F University Yangling Shaanxi China; ^3^ School of Agriculture Ningxia University Yinchuan Ningxia China

**Keywords:** antifungal activity, *Gaeumannomyces graminis*, pyrazolamide, structure‐activity relationships

## Abstract

Fungal infestation in crops can greatly reduce both yield and quality. To discover new fungicides, pyrazol‐5‐yl‐phenylacetamide derivatives were synthesized by elongating the carbon chain of the lead compound pyrazol‐5‐yl‐benzamides. These derivatives were assessed for their effectiveness against fungi. Compound **C‐13** displayed significant antifungal activity against *Sclerotinia sclerotiorum*, with an EC_50_ value of 2.51 mg/L. Additionally, compound **C‐16** showed effective antifungal properties against *Valsa mali*, with an EC_50_ value of 3.14 mg/L. Particularly noteworthy is the superior antifungal efficacy of compound **C‐42** against *Gaeumannomyces graminis*, with an EC_50_ value of 1.81 mg/L, significantly outperforming **Fluxapyroxad** (EC_50_ = 21.01 mg/L). Molecular docking and SDH enzymatic inhibition assay results provided insights into the potential mechanism of action of compound **C‐42**. This study supports the development of pyrazol‐5‐yl‐phenylacetamides as promising fungicides, particularly against *G. graminis*.

## Graphical abstract

The extension of pyrazol‐5‐yl‐benzamides leads to the development of novel phenylacetamides with exceptional efficacy, particularly against *Gaeumannomyces graminis*. Compound **C‐42** demonstrates an EC_50_ of 1.81 mg/L, surpassing the commercial SDHI fluxapyroxad by over tenfold. Enzymatic assays confirm SDH as the specific target. This study highlights pyrazol‐5‐yl‐phenylacetamides as promising fungicides for controlling take‐all disease.

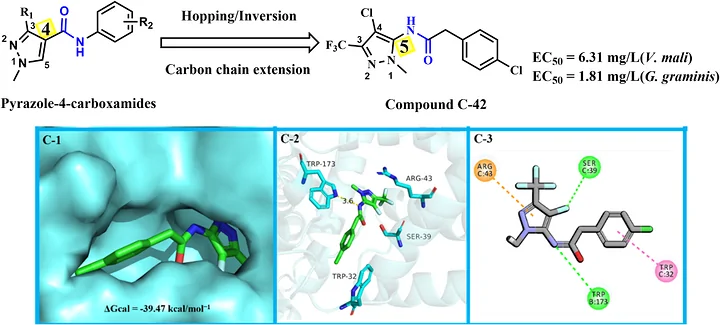

## 1 Introduction

Crop losses caused by phytopathogenic fungi remain a serious challenge in global agriculture [1, 2, 3]. Take‐all, caused by the soilborne fungus *Gaeumannomyces graminis*, is among the most destructive wheat root diseases worldwide, resulting in major losses in yield and quality [4, 5]. Although it continues to be a focus of intensive research, take‐all remains the most important root disease of wheat globally [6]. In recent years, succinate dehydrogenase inhibitors (SDHIs) have attracted considerable interest due to their potent and broad‑spectrum antifungal properties [7, 8, 9, 10, 11, 12, 13]. Currently, 25 SDHI products are commercially available. However, the overuse and inherent target specificity of the single ubiquinone‑binding site [14, 15, 16, 17, 18, 19] have facilitated the emergence of resistant fungal populations [20, 21, 22, 23, 24].

**FIGURE 1:**
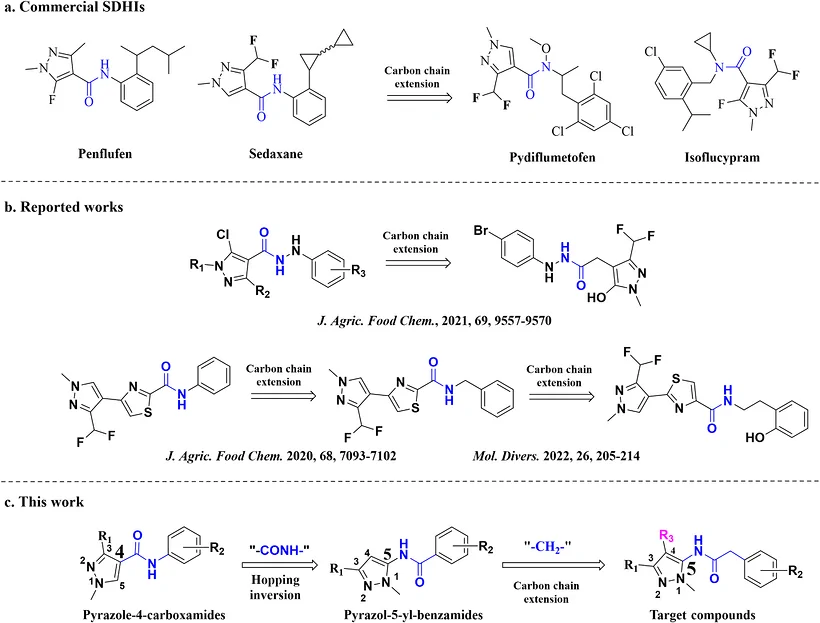
Design strategy of target compounds through carbon chain extension.

In recent years, significant research efforts have focused on the development of innovative SDHIs [25]. Various effective strategies, such as scaffold hopping [26], bioisosterism [27], and virtual screening [16], have been extensively utilized, leading to the discovery of novel and highly potent SDHI candidates. A common approach for lead optimization involves extending the carbon chain to adjust its flexibility and create a target molecule with a non‐planar shape compared to the lead compound (Figure 1a,b) [15, 28, 29, 30]. This modification has the potential to alter the binding mode with the target sites, offering benefits in addressing the escalating issue of resistance and broadening the spectrum of fungal control.

Our previous studies have shown that pyrazol‐5‐yl‐benzamides represent a promising class of lead compounds for SDHI development [31, 32, 33, 34]. In this work, a series of novel pyrazol‐5‐yl‐phenylacetamide derivatives were designed and synthesized utilizing the carbon chain extension strategy (Figure 1c). The *in vitro* antifungal activity of the synthesized compounds was evaluated against six common phytopathogenic fungi/oomycetes, followed by structure–activity relationship (SAR) analysis. To gain insight into the mode of action, scanning electron microscopy (SEM), molecular docking, and SDH enzyme inhibition assays were additionally performed.

## 2 Results and Discussion

### 2.1 Synthesis

Bioactivity‐guided synthesis was utilized to identify fungicidal candidates by investigating the structural features of target compounds. This study was divided into four parts. First, compounds **C‐1**−**C‐16** were synthesized to assess the structure‐activity relationship of the substituents on the phenylacetic acid moiety. These compounds featured a single substitution on the phenylacetic acid moiety and a trifluoromethyl‐substituted pyrazole ring at the third position. Second, compounds **C‐17**−**C‐32** were synthesized to investigate the influence of difluoromethyl substituents at the third position of the pyrazole on the fungicidal activities. The third part involved investigating the effect of multiple substituents or replacement of the phenylacetic acid moiety on the fungicidal activity, leading to the synthesis of compounds **C‐33**−**C‐38**. Last, the fourth part focused on examining the introduction of a fluorine or chlorine atom at the fourth position of the pyrazole ring and determining the combined effect of the substituents at third and fourth positions of the phenylacetic acid moiety on the fungicidal activity, resulting in the synthesis of compounds **C‐39**−**C‐50**.

The synthetic routes for the target compounds **C‐1**−**C‐50** were presented in Scheme 1. The chemical structures of all the synthesized compounds were confirmed by ^1^H NMR, ^13^C NMR, and HRMS analyses, and the related characterization data can be found in Figures S1–S150.

**SCHEME 1:**
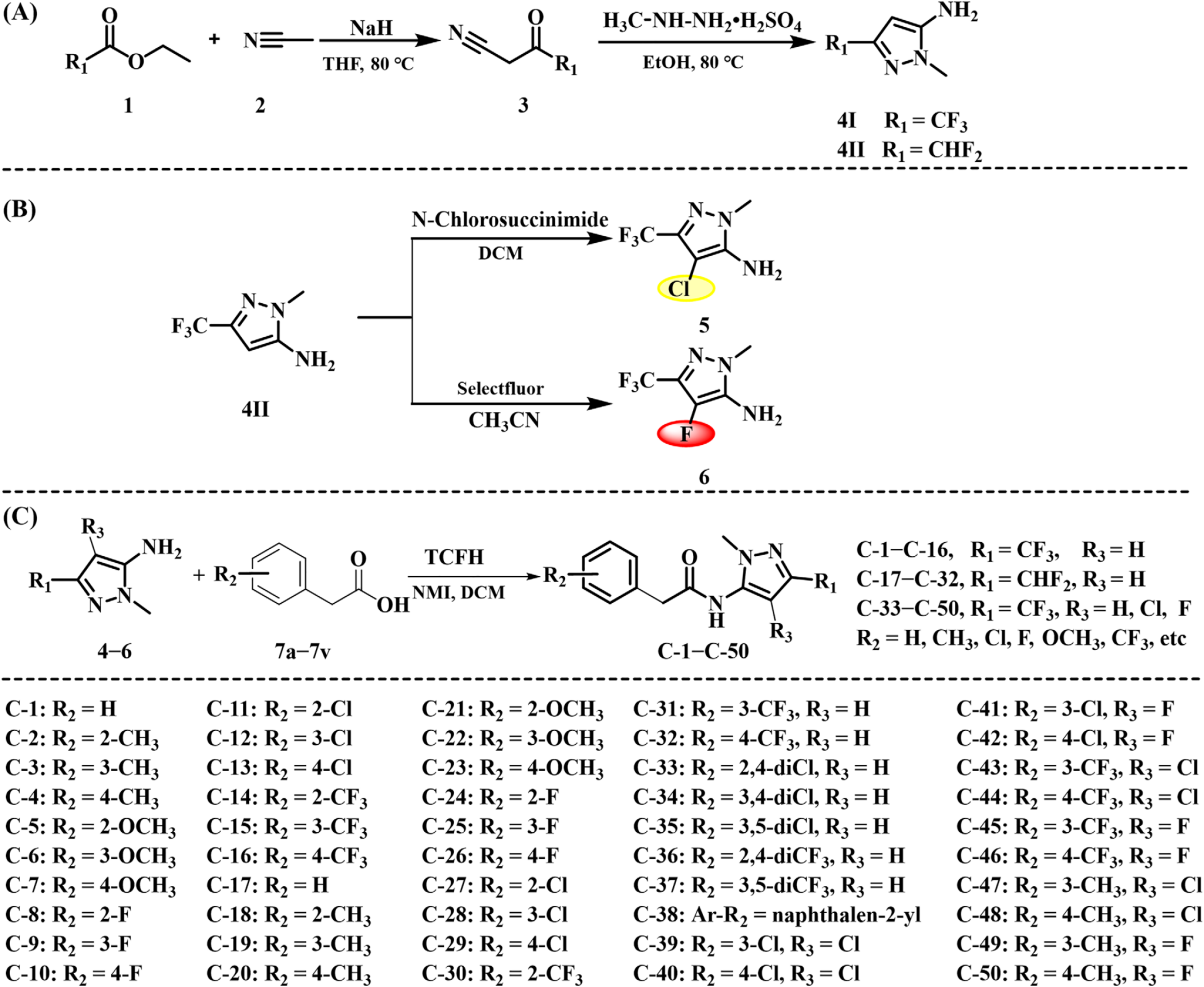
General synthesis of compounds C‐1–C‐50.

### 2.2 *In Vitro* Fungicidal Activities

In order to identify target compounds with effective fungicidal activity, six different substituents (hydrogen, methyl, methoxy, chlorine, fluorine and trifluoromethyl) at three different positions (ortho, meta, and para) on the phenylacetic acid moiety were considered in the design of the target compounds. Consequently, the target compounds **C‐1**−**C‐16** were synthesized, and a preliminary screening was conducted for their fungicidal activity at a concentration of 50 mg/L. The results presented in Table 1 demonstrate significant antifungal activity of several compounds against *Sclerotinia sclerotiorum*, *Valsa mali*, and *G. graminis*. Compounds **C‐13** (R_2_ = 4‐Cl, 92.3%), **C‐15** (R_2_ = 3‐CF_3_, 80.8%), and **C‐16** (R_2_ = 4‐CF_3_, 91.3%) exhibited inhibitory rates exceeding 80% against *S. sclerotiorum*. Similarly, compounds **C‐9** (R_2_ = 3‐F, 80.6%), **C‐13** (R_2_ = 4‐Cl, 80.6%), and **C‐16** (R_2_ = 4‐CF_3_, 83.7%) displayed inhibitory rates surpassing 80% against *V. mali*. Additionally, compounds **C‐12** (R_2_ = 3‐Cl, 96.6%), **C‐13** (R_2_ = 4‐Cl, 98.7%), **C‐15** (R_2_ = 3‐CF_3_, 88.6%), and **C‐16** (R_2_ = 4‐CF_3_, 86.2%) demonstrated inhibitory rates of more than 85% against *G. graminis*. Notably, compounds **C‐13** (R_2_ = 4‐Cl) and **C‐16** (R_2_ = 4‐CF_3_) showed broad‐spectrum antifungal activity. A comparison of the inhibition rates of **C‐11** (R_2_ = 2‐Cl, 69.7%, 62.3%, 44.6%, 44.5%), **C‐12** (R_2_ = 3‐Cl, 79.6%, 73.4%, 96.6%, 73.8%), **C‐13** (R_2_ = 4‐Cl, 92.3%, 80.6%, 98.7%, 71.4%), **C‐14** (R_2_ = 2‐CF_3_, 71.5%, 64.3%, 33.8%, 42.4%), **C‐15** (R_2_ = 3‐CF_3_, 80.8%, 76.0%, 88.6%, 55.7%), and **C‐16** (R_2_ = 4‐CF_3_, 91.3%, 83.7%, 86.2%, 59.5%) against *S. sclerotiorum*, *V. mali, G. graminis*, and *Rhizoctonia solani*, respectively, highlights the favorable activity of target compounds bearing a para‐substituted phenylacetic acid moiety, particularly with chlorine or trifluoromethyl substituents on the phenyl ring.

**TABLE 1:** *In vitro* fungicidal activities (inhibition rate) of compounds **C‐1**−**C‐50** at 50 mg/L ^a^.

Compd.	R_1_	R_2_	R_3_	*S.s*.^b^	*P.c*.^c^	*V. m*.^d^	*B.c*.^e^	*G.g*.^f^	*R.s*.^g^
**C‐1**	CF_3_	H	H	50.9 ± 1.2	25.5 ± 2.0	37.8 ± 1.2	<5	75.2 ± 1.1	36.2 ± 1.6
**C‐2**	CF_3_	2‐CH_3_	H	58.8 ± 0.3	19.3 ± 1.9	50.1 ± 0.6	12.3 ± 2.0	36.8 ± 2.0	31.5 ± 1.2
**C‐3**	CF_3_	3‐CH_3_	H	41.2 ± 1.5	21.2 ± 2.2	32.7 ± 1.5	<5	48.7 ± 1.6	75.7 ± 0.6
**C‐4**	CF_3_	4‐CH_3_	H	61.2 ± 0.9	13.5 ± 1.8	33.7 ± 1.6	35.1 ± 1.3	49.8 ± 1.2	37.6 ± 2.2
**C‐5**	CF_3_	2‐OCH_3_	H	33.3 ± 2.1	31.5 ± 2.3	54.1 ± 1.2	<5	46.3 ± 1.3	29.5 ± 3.1
**C‐6**	CF_3_	3‐OCH_3_	H	55.2 ± 1.6	<5	31.7 ± 1.7	18.3 ± 2.0	53.7 ± 0.5	34.2 ± 0.6
**C‐7**	CF_3_	4‐OCH_3_	H	38.1 ± 1.2	13.7 ± 3.5	37.4 ± 2.0	20.5 ± 1.0	58.1 ± 0.6	31.6 ± 1.1
**C‐8**	CF_3_	2‐F	H	33.9 ± 2.3	<5	36.8 ± 1.6	<5	50.4 ± 1.1	26.5 ± 2.9
**C‐9**	CF_3_	3‐F	H	56.3 ± 0.5	14.5 ± 3.3	80.6 ± 0.7	5.3 ± 2.3	33.8 ± 1.6	33.1 ± 2.0
**C‐10**	CF_3_	4‐F	H	46.1 ± 1.9	<5	39.9 ± 1.2	21.5 ± 3.6	32.2 ± 2.0	26.0 ± 1.3
**C‐11**	CF_3_	2‐Cl	H	69.7 ± 1.2	11.6 ± 2.1	62.3 ± 2.0	31.8 ± 1.6	44.6 ± 1.6	44.5 ± 2.2
**C‐12**	CF_3_	3‐Cl	H	79.6 ± 1.0	44.3 ± 1.5	73.4 ± 1.3	19.4 ± 2.0	96.6 ± 0.1	73.8 ± 1.1
**C‐13**	CF_3_	4‐Cl	H	92.3 ± 0.6	48.1 ± 0.6	80.6 ± 1.2	30.2 ± 2.2	98.7 ± 0.2	71.4 ± 0.4
**C‐14**	CF_3_	2‐CF_3_	H	71.5 ± 0.8	12.6 ± 3.3	64.3 ± 0.6	14.0 ± 1.3	33.8 ± 1.2	42.4 ± 1.3
**C‐15**	CF_3_	3‐CF_3_	H	80.8 ± 1.1	41.2 ± 1.8	76.0 ± 1.2	42.5 ± 2.0	88.6 ± 0.3	55.7 ± 1.2
**C‐16**	CF_3_	4‐CF_3_	H	91.3 ± 0.2	39.5 ± 1.2	83.7 ± 1.3	30.2 ± 2.2	86.2 ± 0.6	59.5 ± 1.6
**C‐17**	CHF_2_	H	H	35.2 ± 2.3	<5	33.7 ± 0.8	<5	48.7 ± 1.1	28.6 ± 2.8
**C‐18**	CHF_2_	2‐CH_3_	H	39.5 ± 2.5	14.5 ± 3.2	24.6 ± 1.1	15.6 ± 2.3	34.7 ± 1.5	22.2 ± 3.1
**C‐19**	CHF_2_	3‐CH_3_	H	42.3 ± 1.6	11.3 ± 2.0	42.5 ± 2.0	<5	32.5 ± 2.2	35.1 ± 1.2
**C‐20**	CHF_2_	4‐CH_3_	H	56.8 ± 0.6	<5	74.5 ± 1.5	<5	66.9 ± 1.3	23.5 ± 1.6
**C‐21**	CHF_2_	2‐OCH_3_	H	44.9 ± 0.7	<5	41.9 ± 1.6	<5	41.3 ± 2.1	12.6 ± 2.1
**C‐22**	CHF_2_	3‐OCH_3_	H	69.7 ± 1.1	12.6 ± 3.3	43.9 ± 1.8	14.5 ± 1.6	17.3 ± 1.9	24.2 ± 3.1
**C‐23**	CHF_2_	4‐OCH_3_	H	35.8 ± 2.1	<5	43.9 ± 1.2	15.6 ± 2.2	58.6 ± 0.8	28.6 ± 3.5
**C‐24**	CHF_2_	2‐F	H	<5	<5	62.3 ± 1.1	<5	34.7 ± 1.3	19.5 ± 2.6
**C‐25**	CHF_2_	3‐F	H	26.7 ± 2.6	<5	27.6 ± 2.0	<5	29.7 ± 2.1	21.2 ± 3.1
**C‐26**	CHF_2_	4‐F	H	<5	<5	40.9 ± 1.6	<5	54.5 ± 0.6	11.4 ± 3.6
**C‐27**	CHF_2_	2‐Cl	H	39.4 ± 1.5	14.5 ± 1.5	21.5 ± 2.0	12.9 ± 1.1	41.3 ± 1.6	26.6 ± 2.6
**C‐28**	CHF_2_	3‐Cl	H	71.3 ± 1.2	12.6 ± 1.3	46.2 ± 0.7	31.5 ± 1.3	28.9 ± 2.2	35.2 ± 2.3
**C‐29**	CHF_2_	4‐Cl	H	64.9 ± 1.0	10.2 ± 1.5	46.0 ± 1.5	23.2 ± 0.6	28.0 ± 1.3	36.6 ± 1.6
**C‐30**	CHF_2_	2‐CF_3_	H	32.1 ± 2.2	13.5 ± 1.2	86.8 ± 0.8	<5	74.4 ± 1.8	28.6 ± 2.2
**C‐31**	CHF_2_	3‐CF_3_	H	12.1 ± 3.5	33.3 ± 2.0	32.3 ± 2.0	26.1 ± 2.3	30.8 ± 2.1	45.2 ± 2.1
**C‐32**	CHF_2_	4‐CF_3_	H	78.2 ± 1.0	25.1 ± 1.8	46.0 ± 1.6	38.9 ± 1.8	46.2 ± 1.3	41.2 ± 1.6
**C‐33**	CF_3_	2,4‐diCl	H	32.8 ± 0.9	25.7 ± 2.3	63.1 ± 1.6	15.3 ± 3.1	30.8 ± 1.2	71.6 ± 2.0
**C‐34**	CF_3_	3,4‐diCl	H	47.0 ± 1.2	35.2 ± 1.6	61.1 ± 2.0	19.8 ± 2.6	40.8 ± 0.9	48.5 ± 1.1
**C‐35**	CF_3_	3,5‐diCl	H	84.3 ± 0.6	56.2 ± 0.8	83.3 ± 1.6	57.7 ± 1.3	45.8 ± 1.6	48.7 ± 0.8
**C‐36**	CF_3_	2,4‐diCF_3_	H	14.1 ± 3.2	23.8 ± 2.0	33.3 ± 2.3	31.5 ± 2.0	33.3 ± 2.0	58.1 ± 1.2
**C‐37**	CF_3_	3,5‐diCF_3_	H	11.1 ± 3.6	33.3 ± 1.5	17.2 ± 2.1	25.2 ± 1.2	28.3 ± 1.9	34.7 ± 1.6
**C‐38**	CF_3_	—	H	<5	21.9 ± 2.0	27.8 ± 1.9	17.1 ± 1.6	28.1 ± 2.1	36.5 ± 2.0
**C‐39**	CF_3_	3‐Cl	Cl	65.7 ± 0.8	47.6 ± 1.1	77.8 ± 0.8	33.3 ± 1.3	55.8 ± 1.3	77.0 ± 1.3
**C‐40**	CF_3_	4‐Cl	Cl	56.1 ± 1.2	69.5 ± 0.6	78.3 ± 0.6	57.7 ± 1.1	98.3 ± 0.2	55.4 ± 1.3
**C‐41**	CF_3_	3‐Cl	F	32.8 ± 2.0	39.1 ± 1.2	63.6 ± 1.2	23.4 ± 0.6	41.7 ± 1.2	85.1 ± 0.9
**C‐42**	CF_3_	4‐Cl	F	60.6 ± 1.5	53.3 ± 0.7	86.9 ± 0.6	35.1 ± 1.3	100	58.1 ± 1.2
**C‐43**	CF_3_	3‐CF_3_	Cl	28.8 ± 2.2	32.4 ± 1.6	43.4 ± 2.3	25.2 ± 1.6	34.2 ± 1.1	74.8 ± 1.1
**C‐44**	CF_3_	4‐CF_3_	Cl	27.8 ± 1.8	35.2 ± 1.5	61.1 ± 0.7	26.1 ± 2.0	53.3 ± 0.9	48.7 ± 0.6
**C‐45**	CF_3_	3‐CF_3_	F	26.9 ± 2.6	22.0 ± 2.0	46.0 ± 1.6	30.8 ± 1.6	33.3 ± 2.1	66.2 ± 0.9
**C‐46**	CF_3_	4‐CF_3_	F	33.8 ± 1.2	29.5 ± 1.6	29.3 ± 2.0	20.7 ± 2.6	30.8 ± 1.3	50.5 ± 1.2
**C‐47**	CF_3_	3‐CH_3_	Cl	51.0 ± 1.6	55.2 ± 0.7	80.8 ± 0.6	45.1 ± 1.3	62.5 ± 0.9	77.0 ± 1.3
**C‐48**	CF_3_	4‐CH_3_	Cl	31.3 ± 2.5	43.8 ± 1.1	71.7 ± 0.7	39.6 ± 2.0	55.8 ± 1.1	56.3 ± 1.2
**C‐49**	CF_3_	3‐CH_3_	F	33.3 ± 2.3	56.2 ± 0.6	62.1 ± 1.6	23.4 ± 1.6	53.3 ± 1.2	92.8 ± 0.2
**C‐50**	CF_3_	4‐CH_3_	F	44.9 ± 1.1	38.0 ± 2.1	32.9 ± 2.0	22.2 ± 2.6	36.7 ± 0.6	45.7 ± 1.2
**Fluxapyroxad**				100	28.9 ± 1.6	59.2 ± 0.9	25.3 ± 2.1	65.0 ± 1.1	89.2 ± 1.6

^a^ Data are given as the mean of triplicate experiments.

^b^ *S. sclerotiorum*.

^c^ *P. capsici*.

^d^ *V. mali*.

^e^ *B. cinerea*.

^f^ *G.graminis*.

^g^ *R. solani*.

To investigate the impact of trifluoromethyl and difluoromethyl substituents at the third position of the pyrazole ring on the biological activity of the target compounds, a series of compounds **C‐17**−**C‐32** bearing difluoromethyl substituents were synthesized and compared with compounds **C‐1**−**C‐16** containing trifluoromethyl substituents. The results revealed that trifluoromethyl substituents on the pyrazole ring could significantly enhance the fungicidal activities of the target compounds. For instance, compound **C‐13** (R_1_ **= **CF_3,_ R_2_ = 4‐Cl) exhibited superior activity compared to **C‐29** (R_1_ **= **CHF_2_, R_2_ = 4‐Cl), while compound **C‐16** (R_1_ **= **CF_3_, R_2_ = 4‐CF_3_) demonstrated higher efficacy than **C‐32** (R_1_ **= **CHF_2_, R_2_ = 4‐CF_3_) against *S. sclerotiorum*, *V. mali, G. graminis*, *Phytophthora capsici*, and *R. solani*.

The screening of the target compounds **C‐1**−**C‐32** initially identified compounds **C‐13** (R_2_ = 4‐Cl) and **C‐16** (R_2_ = 4‐CF_3_) as displaying notable fungicidal activity. Subsequently, to evaluate the impact of polysubstituted phenylacetic acid moiety on fungicidal activity, compounds **C‐33** (R_2_ = 2, 4‐diCl), **C‐34** (R_2_ = 3, 4‐diCl), **C‐35** (R_2_ = 3, 5‐diCl), **C‐36** (R_2_ = 2, 4‐diCF_3_), **C‐37** (R_2_ = 3, 5‐diCF_3_), and **C‐38** (Ar‐R_2_ = naphthalen‐2‐yl) were synthesized. The results indicated that compounds with di‐substituted benzoic acid moiety exhibited significantly lower fungicidal activity compared to those with mono‐substituted benzoic acid moiety. For instance, compounds **C‐33** (R_2_ = 2, 4‐diCl) and **C‐34** (R_2_ = 3, 4‐diCl) showed reduced activity relative to **C‐13** (R_2_ = 4‐Cl), while compounds **C‐36** (R_2_ = 2, 4‐diCF_3_) and **C‐37** (R_2_ = 3, 5‐diCF_3_) demonstrated decreased activity compared to **C‐16** (R_2_ = 4‐CF_3_). Additionally, the replacement of phenylacetic acid moiety in compound **C‐1** with naphthalen‐2‐ylacetic acid moiety resulted in compound **C‐38** (Ar‐R_2_ = naphthalen‐2‐yl) exhibiting a significant decrease in antifungal activity.

The target compounds were further modified by the introduction of fluorine or chlorine atom at the 4‐position of the pyrazole. A series of compounds **C‐39**−**C‐50** containing a 4‐substituted benzoic acid moiety (including chlorine, trifluoromethyl, and methyl substitutions) were designed and synthesized based on the previous experience. The results indicated significant variations in inhibitory effects on different fungi. For *S. sclerotiorum* and *V. mali*, the antifungal activity generally decreased after introducing a fluorine or chlorine atom at the 4‐position of the pyrazole. For example, comparing compound **C‐13** (R_1_ **= **CF_3_, R_2_ = 4‐Cl, R_3_ = H) with compounds **C‐40** (R_1_ **= **CF_3_, R_2_ = 4‐Cl, R_3_ = Cl) and **C‐42** (R_1_ **= **CF_3_, R_2_ = 4‐Cl, R_3_ = F), the antifungal activity decreased. Similarly, when comparing compound **C‐16** (R_1_ **= **CF_3_, R_2_ = 4‐CF_3_, R_3_ = H) with compounds **C‐44** (R_1_ **= **CF_3_, R_2_ = 4‐CF_3_, R_3_ = Cl) and **C‐46** (R_1_ **= **CF_3,_ R_2_ = 4‐CF_3_, R_3_ = F), the antifungal activity also decreased. Conversely, for *G. graminis* and *R. solani*, the antifungal activity in some cases increased after introducing a fluorine or chlorine atom at the 4‐position of the pyrazole. For instance, compound **C‐16** exhibited an inhibition rate of 86.2% against *G. graminis*, which increased to 100% and 98.3% after introducing a fluorine (compound **C‐42**) or chlorine (compound **C‐40**) atom, respectively. Similarly, compound **C‐3** showed an inhibition rate of 75.7% against *R. solani*, which increased to 92.8% after introducing a fluorine atom (compound **C‐49**). However, this series of target compounds demonstrated weaker fungicidal activity against *Botrytis cinerea* and *Phytophthora capsici*.

The preliminary screening assay identified a number of compounds with good fungicidal activity. To further investigate their antifungal activity and structure‐activity relationship, the effective compounds were selected for testing their EC_50_ against *S. sclerotiorum*, *V. mali, G. graminis*, and *R. solani*, as outlined in Table 2. Compound **C‐13** demonstrated the most potent antifungal activity against *S. sclerotiorum* with an EC_50_ of 2.51 mg/L, albeit lower than that of **Fluxapyroxad**. Additionally, compound **C‐16** (R_2_ = 4‐CF_3_, EC_50_ = 3.39 mg/L) exhibited superior efficacy compared to **C‐15** (R_2_ = 3‐CF_3_, EC_50_ = 4.63 mg/L). Particularly noteworthy is that compound **C‐16** displayed significant activity against *V. mali*, with an EC_50_ value of 3.14 mg/L, outperforming **Fluxapyroxad** (EC_50_ = 12.45 mg/L). Compounds **C‐41** (R_2_ = 3‐Cl, R_3_ = F) and **C‐49** (R_2_ = 3‐CH_3_, R_3_ = F) showed EC_50_ values of 4.54 and 2.23 mg/L against *R. solani*, respectively. Notably, the carbon chain extension strategy led to compound **C‑42**, which displayed substantially improved activity against *G. graminis*. It achieved a 100% inhibition rate, far exceeding the 66.2% of the lead compound **9Ig** (Table S1). Moreover, compound **C‐42** (R_2_ = 4‐Cl, R_3_ = F) demonstrated exceptional antifungal activity against *G. graminis*, with an EC_50_ of 1.81 mg/L, surpassing that of **Fluxapyroxad** (EC_50_ = 21.01 mg/L). These results collectively validate the rationality of the carbon chain extension design.

**TABLE 2:** *In vitro* EC_50_ values (mg/L) of the selected compounds.

Fungi	Compd.	EC_50_	95% CI^a^	Regression equation	*R* ^2^
***S. sclerotiorum***	**C‐13**	2.51	1.26–3.86	y = −0.262 + 0.657*x*	0.941
	**C‐15**	4.63	2.94–6.54	y = −0.467 + 0.702*x*	0.931
	**C‐16**	3.39	2.30–4.54	y = −0.488 + 0.920*x*	0.925
	**C‐35**	5.20	4.05–6.48	y = −0.820 + 1.144*x*	0.972
**Fluxapyroxad**		0.55	0.20–0.98	y = 0.253 + 0.974*x*	0.930
*V. mali*	**C‐9**	3.84	2.21–5.63	y = −0.381 + 0.652*x*	0.940
	**C‐13**	5.52	3.96–7.30	y = −0.639 + 0.861*x*	0.962
	**C‐16**	3.14	1.88–4.50	y = −0.226 + 0.397*x*	0.902
	**C‐30**	4.98	3.52–6.63	y = −0.595 + 0.853*x*	0.913
	**C‐35**	6.19	4.30–8.49	y = −0.597 + 0.754*x*	0.928
	**C‐42**	6.31	4.81–8.07	y = −0.790 + 0.987*x*	0.945
	**C‐47**	4.05	2.67–5.55	y = −0.486 + 0.800*x*	0.989
**Fluxapyroxad**		12.45	9.49–18.66	y = −1.243 + 1.135*x*	0.993
*R. solani*	**C‐41**	4.54	3.16–6.07	y = −0.561 + 0.854*x*	0.924
	**C‐49**	2.23	1.37–3.14	y = −0.318 + 0.913*x*	0.948
**Fluxapyroxad**		0.62	0.35–0.88	y = 0.224 + 1.070*x*	0.981
*G. graminis*	**C‐12**	13.88	7.55–65.56	y = −0.487 + 0.426*x*	0.958
	**C‐13**	2.08	0.40–3.85	y = −0.147 + 0.462*x*	0.930
	**C‐15**	6.20	4.04–9.50	y = −0.519 + 0.655*x*	0.976
	**C‐16**	4.57	2.99–6.44	y = −0.497 + 0.753*x*	0.915
	**C‐40**	2.71	1.19–4.25	y = −0.264 + 0.608*x*	0.916
	**C‐42**	1.81	0.64–2.99	y = −0.162 + 0.630*x*	0.901
**Fluxapyroxad**		21.01	17.7–25.8	y = −2.411 + 1.822*x*	0.970

^a^ Confidence interval.

### 2.3 Scanning Electron Microscopy (SEM) Analysis

**FIGURE 2:**
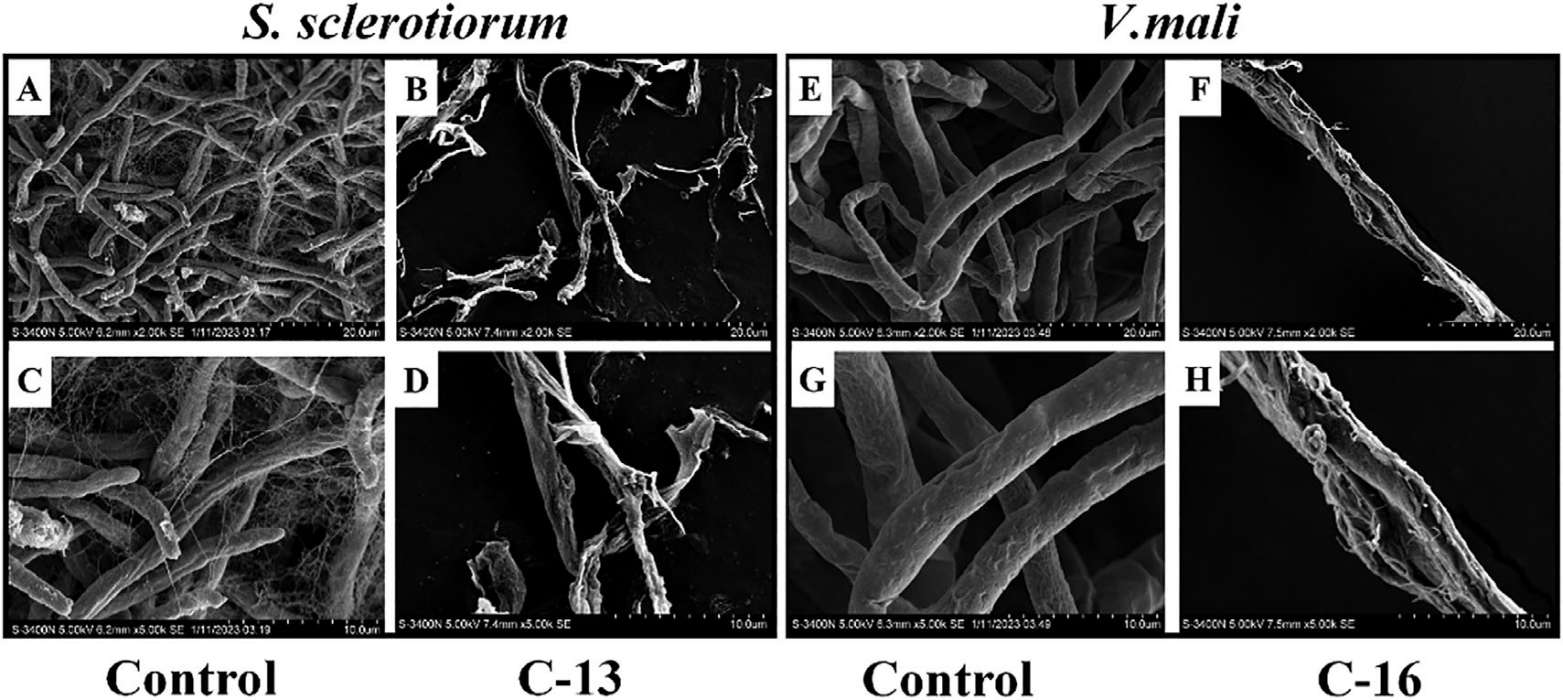
Scanning electron micrographs of the hyphae from the colonies of *S. sclerotiorum* and *V. mali*.

SEM was used to examine the morphological changes in the hyphae of *S. sclerotiorum* and *V. mali* (Figure 2). In the untreated control groups, the hyphae of *S. sclerotiorum* (A, C) and *V. mali* (E, G) appeared regular, smooth, and consistent in thickness and fullness, with a high density. When treated with compounds **C‐13** and **C‐16** at a concentration of 5 mg/L, respectively, a significant decrease in hyphal density was observed for both species. Specifically, the hyphae of *S. sclerotiorum* (B, D) showed surface shrinkage, dry cracks, and extreme unevenness in thickness, while the hyphae of *V. mali* (F, H) displayed wrinkles, severe distortion in individual hyphae, and significant variations in thickness. These results demonstrate that compounds **C‐13** and **C‐16** have detrimental and inhibitory effects on the morphology and growth of *S. sclerotiorum* and *V. mali* hyphae, respectively.

### 2.4 Molecular Docking

**FIGURE 3:**
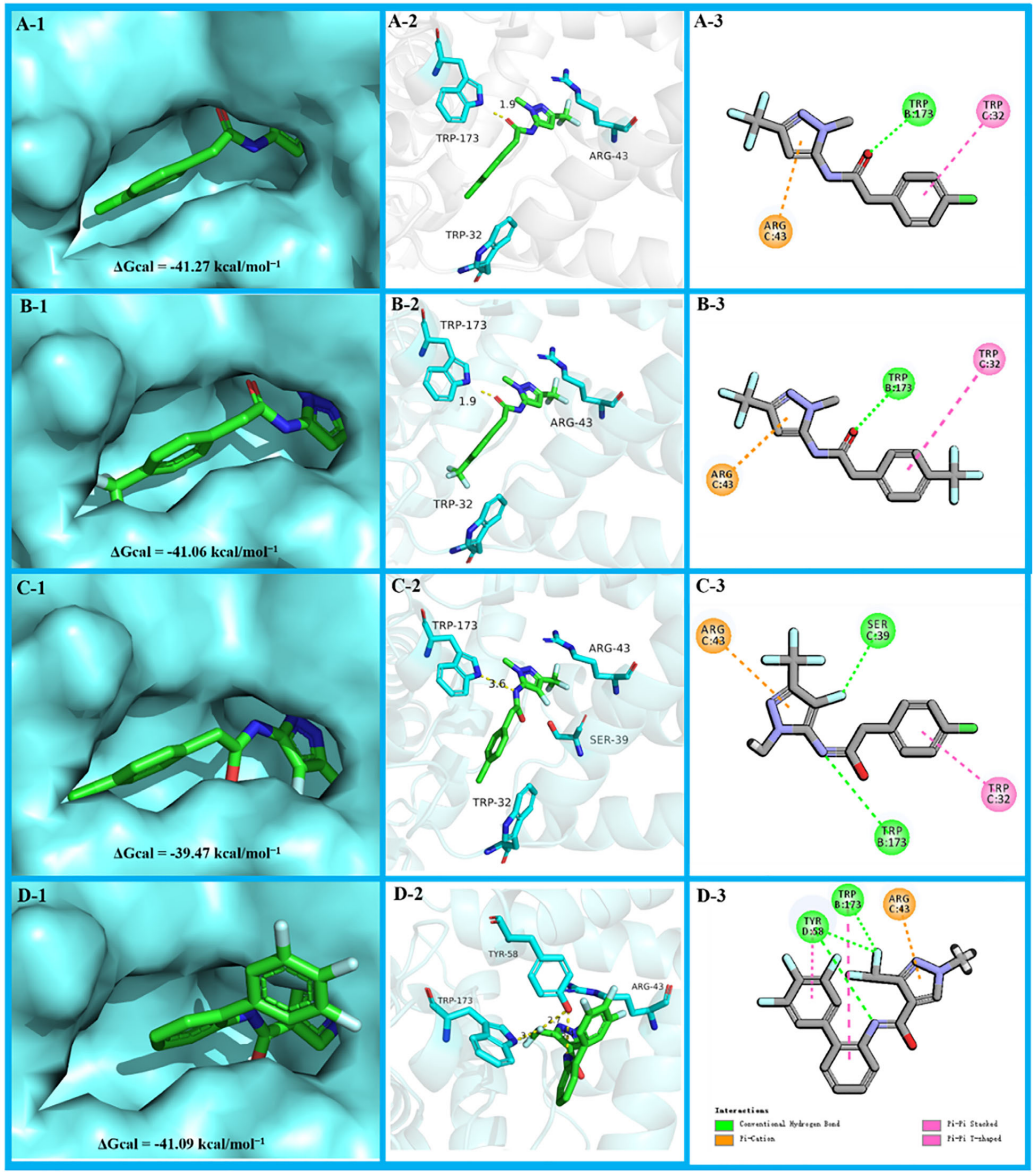
Binding models of **C‐13** (A), **C‐16** (B), **C‐42** (C), and **Fluxapyroxad** (D) in the active site of SDH (PDB entry: 2FBW).

Docking simulations were conducted for compounds **C‑13**, **C‑16**, **C‑42**, and Fluxapyroxad using the crystal structure of SDH (PDB: 2FBW) to probe their binding modes and possible mechanism of action. The results, depicted in Figure 3, revealed that upon carbon chain extension, both the amino and carboxylic acid moieties of the compounds interacted with the active pocket of SDH. Specifically, the phenylacetic acid segments of compounds **C‐13**, **C‐16**, and **C‐42** occupied the left pocket of the active site, while the pyrazole ring section entered the right pocket. In contrast, **Fluxapyroxad** exclusively featured the pyrazole ring interacting with the right pocket of the active site. Additionally, the binding energies of compounds **C‐13**, **C‐16**, **C‐42**, and **Fluxapyroxad** were determined to be −41.27, −41.06, −39.47, and −41.09 kcal/mol, respectively, showing relatively similar values.

As shown in Figure 3 (A2–D2, A3–D3), the tested compounds engage with multiple amino acid residues within the binding pocket, contributing to the stabilization of their binding conformations. All compounds (**C‐13**, **C‐16**, **C‐42**, and **Fluxapyroxad**) engage in p‐π conjugation with Arg 43, enhancing their stability within the binding site. Notably, compounds **C‐13** and **C‐16** form hydrogen bonds with Trp 173 through the oxygen atoms of the amide bond, while compound **C‐42** and **Fluxapyroxad** establish hydrogen bonds with Trp 173 and Tyr 58, respectively, via the nitrogen atom of the amide group. Furthermore, compound **C‐42** forms an additional hydrogen bond between its fluorine atom at the fourth position of the pyrazole ring and Ser 39. Although compounds **C‐13**, **C‐16**, **C‐42**, and **Fluxapyroxad** exhibit similarities in their binding modes with SDH, differences in binding modes and energies are evident among them.

### 2.5 SDH Enzyme Assay *In Vitro*

To gain further insight into its antifungal mechanism, compound **C‐42**, which showed the best *in vitro* activity against *G. Graminis* (EC_50_ = 1.81 mg/L), was evaluated for SDH inhibition. As summarized in Table 3, **C‐42** inhibited SDH with an IC_50_ of 18.33 mg/L, indicating moderate inhibitory activity compared to Fluxapyroxad (IC_50_ = 7.28 mg/L).

**TABLE 3:** SDH inhibition activity (IC_50_).

Compd.	IC_50_ (mg/L)	95% CI^a^	Regression equation	*R* ^2^
**C‐42**	18.33	10.89−41.88	y = −0.767 + 0.608*x*	0.973
**Fluxapyroxad**	7.28	5.18−11.38	y = −0.622 + 0.721*x*	0.994

^a^ Confidence interval.

## 3 Conclusions

In summary, a series of novel pyrazol‐5‐yl‐phenylacetamide derivatives were synthesized and evaluated for their efficacy against various fungi. Several compounds, including **C‐13**, **C‐16**, and **C‐42**, demonstrated significant antifungal activity against *S. sclerotiorum*, *V. mali*, and *G. graminis*, respectively. Notably, compound **C‐42** displayed exceptional antifungal properties against *G. graminis*, with an EC_50_ value of 1.81 mg/L, surpassing **Fluxapyroxad** (EC_50_ = 21.01 mg/L). Molecular docking and SDH enzymatic inhibition assays suggest that these compounds could target SDH. This study provides valuable insights for the potential development of pyrazol‐5‐yl‐phenylacetamides as innovative SDH inhibitors.

### 3.1 Experimental Section

#### 3.1.1 Materials and Instruments

All reagent‐grade chemicals were procured from commercial sources such as Energy and Adamas. ^1^H, and ^13^C NMR spectra were recorded on a Bruker AV‐400 Hz spectrometer, with CDCl_3_ or DMSO‐*d*_6_ as the solvent and tetramethylsilane as the internal standard. Chemical shifts were reported in ppm (δ). ESI–MS spectra were obtained using a Mariner System 5304 mass spectrometer. Melting points (mp) were measured using a WRS‐1B melting point apparatus (Jingsong, Shanghai, China) without correction. A scanning electron microscope (Hitachi, S‐3400 N, Tokyo, Japan) was employed to observe the microscopic morphology of fungal hyphae. Conductivity was measured by a CON510 Eutech/Oakton conductometer (OAKTON Instruments, Waltham).

#### 3.1.2 Chemistry

The synthetic routes of the target compounds **C**‐**1**–**C**‐**50** are shown in Scheme 1. Intermediates **3** and **4** were synthesized from acetonitrile and a substituted ethyl acetate [32], while intermediates **5** and **6** were prepared according to the literature method [33].

##### 3.1.2.1 General Procedure for the Synthesis of Target Compound **C**‐**1**–**C**‐**50**

Following the methodology described in reference [35], a reaction was performed by dissolving a substituted 2‐phenylacetic acid (1.1 mmol) and intermediate **4I**, **4II**, **5**, or **6** (1 mmol) in 3 mL of dichloromethane (DCM). Subsequently, *N*‐methylimidazole (3.5 mmol) and *N*,*N*,*N'*,*N'*‐tetramethylchloroformamidinium‐hexafluorophosphate (TCFH, 1.2 mmol) were added to the solution. The reaction mixture was then stirred at room temperature for 4−20 h, and the resulting solution was concentrated to yield the crude product. This crude product was then purified by silica gel chromatography (PE/EtOAc = 5:1) to afford the corresponding target compounds **C**‐**1**–**C**‐**50**.

### 3.2 Antifungal Bioassay *In Vitro*

The fungicidal activities of all synthesized compounds were assessed *in vitro* against *B. cinerea*, *V. mali*, *R. solani*, *S. sclerotiorum*, *P. capsici*, and *G. graminis* at a concentration of 50 mg/L using the mycelia growth inhibition method [32, 36, 37] for preliminary screening. The detailed experimental method is available in the Supporting Information.

### 3.3 SEM Analysis

The SEM assay was carried out following the previously described method [32, 36].

### 3.4 Molecular Docking

Molecular docking was accomplished by employing a method that has been reported [32, 34].

### 3.5 *In Vitro* SDH Enzyme Assay

The SDH enzyme activities of **C‐42** and **Fluxapyroxad** were determined using the Mlbio succinate dehydrogenase assay kit (ml076573) and evaluated according to a previously established protocol [32].

## Author Contributions

Dan Xu conceived and designed the study, provided overall supervision, and administered the project. Guo‑Tai Lin and Wei Wang contributed to the methodology together with Xi‑Ze Zhang and Li Luo, and also collaborated on data curation and validation. Guo‑Tai Lin and Wei Wang performed the formal analysis, investigation, and software implementation, and were primarily responsible for the visualization of the data and the writing of the original draft. The manuscript was critically reviewed and edited by Xi‑Ze Zhang, Li Luo, Gong Xu, and Dan Xu. Resources were provided by Gong Xu and Dan Xu, while funding acquisition was secured by Wei Wang, Li Luo, and Dan Xu. All authors have read and approved the final manuscript.

## Conflicts of Interest

The authors declare no conflicts of interest.

## Supporting information




**Supporting File 1**: cbdv71784‐sup‐0001‐SuppMat.docx

## Data Availability

The data supporting the findings of this study are available in the Supporting Information of this article, including: ^1^H NMR, ^1^ ^3^C NMR, ^1^ ^9^F NMR, and HRMS (ESI) spectra of the target compounds (Figures S1–S150); physical data of the target compounds; *in vitro* fungicidal activity assay methods; and the activity data of the lead compound 9Ig against plant pathogens (Table S1).

## References

[1] H. C. J. Godfray , J. R. Beddington , I. R. Crute , et al., “Food Security: The Challenge of Feeding 9 Billion People,” Science 327 (2010): 812–818, 10.1126/science.1185383. 20110467

[2] G. Caioni , C. Merola , M. Perugini , et al., “An Experimental Approach to Study the Effects of Realistic Environmental Mixture of Linuron and Propamocarb on Zebrafish Synaptogenesis,” International Journal of Environmental Research and Public Health 18 (2021): 4664, 10.3390/ijerph18094664. PMC8124988 33925709

[3] V. S. Brauer , C. P. Rezende , A. M. Pessoni , et al., “Antifungal Agents in Agriculture: Friends and Foes of Public Health,” Biomolecules 9 (2019): 521, 10.3390/biom9100521. PMC6843326 31547546

[4] L. H. Xie , X. Quan , J. Zhang , et al., “Selection of Reference Genes for Real‐Time Quantitative PCR Normalization in the Process of *Gaeumannomyces graminis* Var. Tritici Infecting Wheat,” Plant Pathology Journal 35 (2019): 11–18, 10.5423/PPJ.OA.03.2018.0038. PMC6385657 30828275

[5] Y. N. Cheng , L. Sun , H. Meng , et al., “Structure–Activity Studies of *N*‐Heterocyclic Benzoyl Arylamine Derivatives Led to a Highly Fungicidal Candidate Against *Gaeumannomyces graminis* Var. Tritici and Four Fusarium Wheat Pathogens,” Journal of Agricultural and Food Chemistry 70 (2022): 10305–10315, 10.1021/acs.jafc.2c03455. 35950372

[6] Z. Wang , Q. Peng , X. Gao , et al., “Novel Fungicide 4‐Chlorocinnamaldehyde Thiosemicarbazide (PMDD) Inhibits Laccase and Controls the Causal Agent of Take‐All Disease in Wheat, *Gaeumannomyces graminis* Var. Tritici,” Journal of Agricultural and Food Chemistry 68 (2020): 5318–5326, 10.1021/acs.jafc.0c01260. 32356426

[7] X. L. Zhu , L. Xiong , H. Li , X. Y. Song , J. J. Liu , and G. F. Yang , “Computational and Experimental Insight Into the Molecular Mechanism of Carboxamide Inhibitors of Succinate‐Ubquinone Oxidoreductase,” ChemMedChem 9 (2014): 1512–1521, 10.1002/cmdc.201300456. 24678033

[8] L. Xiong , X.‐L. Zhu , Y.‐Q. Shen , W. K. W. M. Wishwajith , K. Li , and G.‐F. Yang , “Discovery of N‐Benzoxazol‐5‐yl‐Pyrazole‐4‐Carboxamides as Nanomolar SQR Inhibitors,” European Journal of Medicinal Chemistry 95 (2015): 424–434, 10.1016/j.ejmech.2015.03.060. 25841198

[9] L. Xiong , X. L. Zhu , H. W. Gao , et al., “Discovery of Potent Succinate‐Ubiquinone Oxidoreductase Inhibitors via Pharmacophore‐Linked Fragment Virtual Screening Approach,” Journal of Agricultural and Food Chemistry 64 (2016): 4830–4837, 10.1021/acs.jafc.6b00325. 27225833

[10] X. H. Lv , Z. L. Ren , P. Liu , et al., “Design, Synthesis and Biological Evaluation of Novel Nicotinamide Derivatives Bearing a Substituted Pyrazole Moiety as Potential SDH Inhibitors,” Pest Management Science 73 (2017): 1585–1592, 10.1002/ps.4488. 27860139

[11] H. Li , M. Q. Gao , Y. Chen , Y. X. Wang , X. L. Zhu , and G. F. Yang , “Discovery of Pyrazine‐Carboxamide‐Diphenyl‐Ethers as Novel Succinate Dehydrogenase Inhibitors via Fragment Recombination,” Journal of Agricultural and Food Chemistry 68 (2020): 14001–14008, 10.1021/acs.jafc.0c05646. 33185088

[12] H. Sierotzki and G. Scalliet , “A Review of Current Knowledge of Resistance Aspects for the Next‐Generation Succinate Dehydrogenase Inhibitor Fungicides,” Phytopathology® 103 (2013): 880–887, 10.1094/PHYTO-01-13-0009-RVW. 23593940

[13] C. E. Keohane , A. D. Steele , C. Fetzer , et al., “Promysalin Elicits Species‐Selective Inhibition of Pseudomonas aeruginosa by Targeting Succinate Dehydrogenase,” Journal of the American Chemical Society 140 (2018): 1774–1782, 10.1021/jacs.7b11212. PMC5869686 29300464

[14] Z. Wu , H.‐Y. Park , D. Xie , et al., “Synthesis, Biological Evaluation, and 3D‐QSAR Studies of N ‐(Substituted Pyridine‐4‐yl)‐1‐(Substituted Phenyl)‐5‐Trifluoromethyl‐1 H ‐Pyrazole‐4‐Carboxamide Derivatives as Potential Succinate Dehydrogenase Inhibitors,” Journal of Agricultural and Food Chemistry 69 (2021): 1214–1223, 10.1021/acs.jafc.0c05702. 33480684

[15] J. J. Zhu , P. Y. Wang , Z. Q. Long , et al., “Design, Synthesis, and Biological Profiles of Novel 1,3,4‐Oxadiazole‐2‐Carbohydrazides With Molecular Diversity,” Journal of Agricultural and Food Chemistry 70 (2022): 2825–2838, 10.1021/acs.jafc.1c07190. 35201749

[16] L. Xiong , H. Li , L. N. Jiang , et al., “Structure‐Based Discovery of Potential Fungicides as Succinate Ubiquinone Oxidoreductase Inhibitors,” Journal of Agricultural and Food Chemistry 65 (2017): 1021–1029, 10.1021/acs.jafc.6b05134. 28110534

[17] G. Wei , M. W. Huang , W. J. Wang , et al., “Expanding the Chemical Space of Succinate Dehydrogenase Inhibitors via the Carbon–Silicon Switch Strategy,” Journal of Agricultural and Food Chemistry 69 (2021): 3965–3971, 10.1021/acs.jafc.0c07322. 33779164

[18] X. Hua , W. Liu , Y. Su , et al., “Studies on the Novel Pyridine Sulfide Containing SDH Based Heterocyclic Amide Fungicide,” Pest Management Science 76 (2020): 2368–2378, 10.1002/ps.5773. 32022382

[19] B. Yu , B. Zhao , Z. Hao , et al., “Design, Synthesis and Biological Evaluation of Pyrazole‐Aromatic Containing Carboxamides as Potent SDH Inhibitors,” European Journal of Medicinal Chemistry 214 (2021): 113230, 10.1016/j.ejmech.2021.113230. 33581553

[20] Y. Zhao , A. Zhang , X. Wang , K. Tao , H. Jin , and T. Hou , “Novel Pyrazole Carboxamide Containing a Diarylamine Scaffold Potentially Targeting Fungal Succinate Dehydrogenase: Antifungal Activity and Mechanism of Action,” Journal of Agricultural and Food Chemistry 70 (2022): 13464–13472, 10.1021/acs.jafc.2c00748. 36250688

[21] A. Zhang , Y. Yue , J. Yang , et al., “Design, Synthesis, and Antifungal Activities of Novel Aromatic Carboxamides Containing a Diphenylamine Scaffold,” Journal of Agricultural and Food Chemistry 67 (2019): 5008–5016, 10.1021/acs.jafc.9b00151. 30977370

[22] F. Wen , H. Jin , K. Tao , and T. Hou , “Design, Synthesis and Antifungal Activity of Novel Furancarboxamide Derivatives,” European Journal of Medicinal Chemistry 120 (2016): 244–251, 10.1016/j.ejmech.2016.04.060. 27191618

[23] W. Yan , X. Wang , K. Li , et al., “Design, Synthesis, and Antifungal Activity of Carboxamide Derivatives Possessing 1,2,3‐Triazole as Potential Succinate Dehydrogenase Inhibitors,” Pesticide Biochemistry and Physiology 156 (2019): 160–169, 10.1016/j.pestbp.2019.02.017. 31027576

[24] B. Luo and Y. Ning , “Comprehensive Overview of Carboxamide Derivatives as Succinate Dehydrogenase Inhibitors,” Journal of Agricultural and Food Chemistry 70 (2022): 957–975, 10.1021/acs.jafc.1c06654. 35041423

[25] T. Du , S. Lu , Z. Zhu , et al., “Pyrazole Derivatives: Recent Advances in Discovery and Development of Pesticides,” Chinese Chemical Letters 36 (2025): 110912, 10.1016/j.cclet.2025.110912.

[26] S. Sun , L. Chen , J. Huo , et al., “Discovery of Novel Pyrazole Amides as Potent Fungicide Candidates and Evaluation of Their Mode of Action,” Journal of Agricultural and Food Chemistry 70 (2022): 3447–3457, 10.1021/acs.jafc.2c00092. 35282681

[27] X. Cheng , Z. Xu , H. Luo , X. Chang , and X. Lv , “Design, Synthesis, and Biological Evaluation of Novel Pyrazol‐5‐yl‐Benzamide Derivatives Containing Oxazole Group as Potential Succinate Dehydrogenase Inhibitors,” Journal of Agricultural and Food Chemistry 70 (2022): 13839–13848, 10.1021/acs.jafc.2c04708. 36270026

[28] X. Wang , M. Wang , L. Han , et al., “Novel Pyrazole‐4‐Acetohydrazide Derivatives Potentially Targeting Fungal Succinate Dehydrogenase: Design, Synthesis, Three‐Dimensional Quantitative Structure–Activity Relationship, and Molecular Docking,” Journal of Agricultural and Food Chemistry 69 (2021): 9557–9570, 10.1021/acs.jafc.1c03399. 34382800

[29] Z. S. Hao , B. Yu , W. Gao , et al., “Design, Synthesis and Fungicidal Activity of Pyrazole–Thiazole Carboxamide Derivatives,” Molecular Diversity 26 (2022): 205–214, 10.1007/s11030-020-10177-0. 33792811

[30] X.‐H. Liu , L. Qiao , Z.‐W. Zhai , et al., “Novel 4‐Pyrazole Carboxamide Derivatives Containing Flexible Chain Motif: Design, Synthesis and Antifungal Activity,” Pest Management Science 75 (2019): 2892–2900, 10.1002/ps.5463. 31050111

[31] W. Wang , X. J. Liu , G. T. Lin , J. P. Wu , G. Xu , and D. Xu , “Novel N ‐(1 H ‐Pyrazol‐5‐yl)Nicotinamide Derivatives: Design, Synthesis and Antifungal Activity,” Chemistry & Biodiversity 19 (2022): e202101032, 10.1002/cbdv.202101032. 35275425

[32] W. Wang , J. Wang , F. Wu , H. Zhou , D. Xu , and G. Xu , “Synthesis and Biological Activity of Novel Pyrazol‐5‐yl‐Benzamide Derivatives as Potential Succinate Dehydrogenase Inhibitors,” Journal of Agricultural and Food Chemistry 69 (2021): 5746–5754, 10.1021/acs.jafc.0c08094. 33988994

[33] W. Wang , J. Wang , J. Wu , et al., “Rational Design, Synthesis, and Biological Evaluation of Fluorine‐ and Chlorine‐Substituted Pyrazol‐5‐yl‐Benzamide Derivatives as Potential Succinate Dehydrogenase Inhibitors,” Journal of Agricultural and Food Chemistry 70 (2022): 7566–7575, 10.1021/acs.jafc.2c01901. 35674516

[34] W. Wang , F. Wu , Y. Ma , D. Xu , and G. Xu , “Study on Synthesis and Antifungal Activity of Novel Benzamides Containing Substituted Pyrazole Unit,” Chinese Journal of Organic Chemistry 42 (2022): 607, 10.6023/cjoc202108009.

[35] G. L. Beutner , I. S. Young , M. L. Davies , et al., “TCFH–NMI: Direct Access to N ‐Acyl Imidazoliums for Challenging Amide Bond Formations,” Organic Letters 20 (2018): 4218–4222, 10.1021/acs.orglett.8b01591. 29956545

[36] W. Wang , S. Zhang , J. H. Wang , F. R. Wu , T. Wang , and G. Xu , “Bioactivity‐Guided Synthesis Accelerates the Discovery of 3‐(Iso)Quinolinyl‐4‐Chromenones as Potent Fungicide Candidates,” Journal of Agricultural and Food Chemistry 69 (2021): 491–500, 10.1021/acs.jafc.0c06700. 33382606

[37] W. Wang , X. Cheng , X. Cui , D. G. Xia , Z. Q. Wang , and X. H. Lv , “Synthesis and Biological Activity of Novel Pyrazolo[3,4‐d]Pyrimidin‐4‐One Derivatives as Potent Antifungal Agent,” Pest Management Science 77 (2021): 3529–3537, 10.1002/ps.6406. 33837653

