# Supplementary material for: Design, Synthesis, and Biological Evaluation of Pyrazol‐5‐yl‐Phenylacetamide Derivatives as Potential Fungicidal Agents
Source: Chem Biodivers. 2026 Sep 27;23(9):e71784. doi: 10.1002/cbdv.71784 (PMC13616471; doi:10.1002/cbdv.71784)
Supplement: Supplementary file 1 [file CBDV-23-e71784-s001.docx]

**Design, Synthesis, and Biological Evaluation of Pyrazol-5-yl-phenylacetamide Derivatives as Potential Fungicidal Agents**

Guo-Tai Lin,^#,a,b^ Wei Wang,^#,b,c^ Xi-Zhe Zhang,^b^ Li Luo,^b^ Gong Xu,^a,b^ and Dan Xu^*,^ ^a,b^

**Supporting Information**

^a^ Shaanxi Key Laboratory of Natural Products & Chemical Biology, College of Chemistry & Pharmacy, Northwest A&F University, Yangling 712100, Shaanxi, China,

^b^ Key Laboratory of Plant Protection Resources and Pest Management of Ministry of Education, College of Plant Protection, Northwest A&F University, Yangling 712100, Shaanxi, China

^c^School of Agriculture, Ningxia University, Yinchuan 750021, Ningxia, China

^#^ Guo-Tai Lin and Wei Wang contributed equally

* Corresponding author: Dr. Dan Xu, orcid.org/0000-0002-0456-3893, E-mail: danxu@nwafu.edu.cn

**List of Supplementary Materials**

**Page 3:** Biological Assay

**Page 3: Table S1:**The activity data of lead compound **9Ig** against plant pathogens

**Page 4−15:** The Physical Data of Target Compounds

**Page 16−90:** ^1^H NMR, ^13^C NMR, ^19^F NMR, and HRMS (ESI) Spectra of the Target Compounds

**Biological Assay**

**Fungi.**

*Botrytis cinerea, Valsa mali, Rhizoctonia solani, Sclerotinia sclerotiorum, Phytophthora capsici,* and *Gaeumannomyces graminis* were provided by the College of Plant Protection, Northwest A&F University.

***In Vitro* Fungicidal Activities.**

The tested compounds were dissolved in DMSO to give a 10 mg/mL stock solution before mixing with PDA. The media containing the corresponding compounds at a concentration of 20 mg/L was then poured into sterilized Petri dishes for primary screening. After 24**−**72 h at 28 °C, the colony diameter of each strain was measured. The percentage inhibition rate was calculated as (C-A)/(C-B) × 100%, where A represents the colony diameter in the Petri dishes with the tested compounds, B represents the diameter of the mycelial disc, and C is the mean colony diameter in the control Petri dishes. **Fluxapyroxad** was selected as the positive control. Each treatment was performed three times. The inhibition rate of the potent compounds was further tested, and the corresponding EC_50_ values were calculated using SPSS 24.0.

**The activity data of lead compound 9Ig against plant pathogens.**

**Table S1.** *In vitro* fungicidal activities (inhibition rate) of compound **9Ig** at 50 mg/L.^a^

| **Compd.** | R_1_ | R_2_ | R_3_ | *S.s^b^* | *P.c^c^* | *V. m^d^* | *B.c^e^* | *G.g^f^* | *R.s^g^* |
| --- | --- | --- | --- | --- | --- | --- | --- | --- | --- |
| **9Ig** | CF_3_ | 4-Cl | F | 93.2 ± 0.3 | 35.5 ± 2.0 | 89.7 ± 0.2 | 62.1 ± 0.7 | **66.2 ± 1.8** | 44.3 ± 1.4 |
| **C-42** | CF_3_ | 4-Cl | F | 60.6 ± 1.5 | 54.3 ± 0.7 | 86.9 ± 0.6 | 35.1 ± 1.3 | **100** | 58.1 ± 1.2 |

^a^ Data are given as the mean of triplicate experiments; *S.s.*^b^ *S. sclerotiorum*; *P.c*.^c^ *P. capsici*; *V.m*.^d^ *V. mali*; *B.c*.^e^ *B. cinerea;* *G.g.*^f^*G.graminis*; *R.s.* ^g^ *R. solani.*

**The Physical Data of Compounds C-1−C-50**

*N-(1-methyl-3-(trifluoromethyl)-1H-pyrazol-5-yl)-2-phenylacetamide* (**C-1**)

Yellow solid, yield 72%; mp = 120.0**–**122.8 ℃; ^1^H NMR (400 MHz, DMSO-*d*_6_) δ 10.50 (s, 1H), 7.35 (d, *J* = 5.0 Hz, 4H), 7.27 (dq, *J* = 8.7, 4.2 Hz, 1H), 6.67 (s, 1H), 3.77 (s, 3H), 3.75 (s, 2H); ^13^C NMR (101 MHz, DMSO-*d*_6_) δ 169.8, 139.2 (q, *J* = 34.6 Hz), 135.7, 129.7, 121.8 (q, *J* = 267.8 Hz, 97.3, 42.5, 36.9; ESI-MS calculated for C_13_H_12_F_3_N_3_ONa [M+Na]^+^, 306.0825; found 306.0822.

*N-(1-methyl-3-(**trifluoromethyl)-1H-pyrazol-5-yl)-2-(o-tolyl)acetamide* (**C-2**)

White solid, yield 81%; mp = 143.3**–**145.3 ℃; ^1^H NMR (400 MHz, DMSO-*d*_6_) δ 10.45 (s, 1H), 7.25 (s, 1H), 7.17 (t, *J* = 5.5 Hz, 3H), 6.65 (s, 1H), 3.78 (s, 5H), 2.29 (s, 3H); ^13^C NMR (101 MHz, DMSO-*d*_6_) δ 169.7, 139.3 (q, *J* = 37.2 Hz), 138.6, 137.2, 134.4, 130.5, 127.4, 126.3, 125.8, 121.8 (q, *J* = 268.1 Hz), 97.4, 36.9, 35.2, 19.8; ESI-MS calculated for C_14_H_14_F_3_N_3_ONa [M+Na]^+^, 320.0981; found 320.0983.

*N-(1-methyl-3-(trifluoromethyl)-1H-pyrazol-5-yl)-2-(m-tolyl)acetamide* (**C-3**)

Yellow solid, yield 76%; mp = 114.0**–**116.6 ℃; ^1^H NMR (400 MHz, DMSO-*d*_6_) δ 10.44 (s, 1H), 7.22 (t, *J* = 7.5 Hz, 1H), 7.18–7.11 (m, 2H), 7.07 (d, *J* = 7.4 Hz, 1H), 6.65 (s, 1H), 3.76 (s, 3H), 3.70 (s, 2H), 2.30 (s, 3H); ^13^C NMR (101 MHz, DMSO-*d*_6_) δ 169.8, 139.2 (q, *J* = 37.4 Hz), 138.6, 137.9, 135.5, 130.3, 128.7, 127.8, 126.7, 121.8 (q, *J* = 268.1 Hz), 97.3, 42.4, 36.9, 21.4; ESI-MS calculated for C_14_H_14_F_3_N_3_ONa [M+Na]^+^, 320.0981; found 320.0980.

*N-(1-methyl-3-(trifluoromethyl)-1H-pyrazol-5-yl)-2-(p-tolyl)acetamide* (**C-4**)

Yellow solid, yield 75%; mp = 128.8**–**131.3 ℃; ^1^H NMR (400 MHz, DMSO-*d*_6_) δ 10.41 (s, 1H), 7.22 (d, *J* = 6.8 Hz, 2H), 7.15 (d, *J* = 7.1 Hz, 2H), 6.64 (s, 1H), 3.75 (s, 3H), 3.68 (s, 2H), 2.29 (s, 3H); ^13^C NMR (101 MHz, DMSO-*d*_6_) δ 170.0, 138.6, 136.2, 132.6, 129.5, 129.4, 123.1, 97.4, 42.1, 36.9, 21.1; ESI-MS calculated for C_14_H_14_F_3_N_3_ONa [M+Na]^+^, 320.0981; found 320.0981.

*N-(3-(trifluoromethyl)-1-methyl-1H-pyrazol-5-yl)-2-(2-methoxyphenyl)acetamide* (**C-5**)

White solid, yield 62%; mp = 104.0**–**107.8 ℃; ^1^H NMR (400 MHz, DMSO-*d*_6_) δ 10.33 (s, 1H), 7.25 (q, *J* = 7.7 Hz, 2H), 6.99 (d, *J* = 8.1 Hz, 1H), 6.91 (t, *J* = 7.4 Hz, 1H), 6.62 (s, 1H), 3.78 (s, 6H), 3.70 (s, 2H); ^13^C NMR (101 MHz, DMSO-*d*_6_) δ 169.9, 157.7, 139.2 (d, *J* = 39.2 Hz), 138.8, 131.5, 128.8, 124.0, 120.7, 121.8 (q, *J* = 268.1 Hz), 111.1, 97.5, 55.8, 37.4, 36.8; ESI-MS calculated for C_14_H_14_F_3_N_3_O_2_Na [M+Na]^+^, 336.0930; found 336.0929.

*N-(3-(trifluoromethyl)-1-methyl-1H-pyrazol-5-yl)-2-(3-methoxyphenyl)acetamide* (**C-6**)

Yellow solid, yield 80%; mp = 108.8**–**112.5 ℃; ^1^H NMR (400 MHz, DMSO-*d*_6_) δ 10.44 (s, 1H), 7.25 (t, *J* = 8.0 Hz, 1H), 6.91 (d, *J* = 7.1 Hz, 2H), 6.84 (dd, *J* = 7.3, 1.9 Hz, 1H), 6.65 (s, 1H), 3.76 (s, 3H), 3.75 (s, 3H), 3.71 (s, 2H); ^13^C NMR (101 MHz, DMSO-*d*_6_) δ 169.6, 159.7, 139.2 (q, *J* = 37.4 Hz), 138.6, 137.1, 129.8, 121.8, 121.8 (q, *J* = 268.1 Hz), 115.4, 112.6, 97.4, 55.4, 42.5, 36.9; ESI-MS calculated for C_14_H_14_F_3_N_3_O_2_Na [M+Na]^+^, 336.0930; found 336.0932.

*N-(3-(trifluoromethyl)-1-methyl-1H-pyrazol-5-yl)-2-(4-methoxyphenyl)acetamide* (**C-7**)

Yellow solid, yield 69%; mp = 119.2**–**121.0 ℃; ^1^H NMR (400 MHz, Chloroform-*d*) δ 7.26 (s, 1H), 7.23 (s, 1H), 7.10 (s, 1H), 6.95 (d, *J* = 8.5 Hz, 2H), 6.47 (s, 1H), 3.83 (s, 3H), 3.71 (s, 2H), 3.61 (s, 3H); ^13^C NMR (101 MHz, Chloroform-*d*) δ 169.8, 159.4, 140.9 (q, *J* = 38.6 Hz), 136.0, 130.6, 125.3, 120.9 (q, *J* = 268.5 Hz), 114.9, 98.6, 55.4, 42.8, 36.1; ESI-MS calculated for C_14_H_14_F_3_N_3_O_2_Na [M+Na]^+^, 336.0930; found 336.0931.

*N-(3-(trifluoromethyl)-1-methyl-1H-pyrazol-5-yl)-2-(2-fluorophenyl)acetamide* (**C-8**)

White solid, yield 88%; mp = 153.7**–**155.8 ℃; ^1^H NMR (400 MHz, DMSO-*d*_6_) δ 10.53 (s, 1H), 7.37 (dt, *J* = 29.3, 6.8 Hz, 2H), 7.19 (q, *J* = 7.2 Hz, 2H), 6.65 (s, 1H), 3.83 (s, 2H), 3.79 (s, 3H); ^13^C NMR (101 MHz, DMSO-*d*_6_) δ 168.8, 162.4, 159.9, 139.3 (q, *J* = 37.2 Hz), 138.5, 132.5, 132.5, 129.5, 129.4, 124.7, 123.1, 122.8, 121.8 (q, *J* = 268.1 Hz), 115.6, 115.4, 36.9, 35.8; ESI-MS calculated for C_13_H_11_F_4_N_4_ONa [M+Na]^+^, 324.0731; found 324.0728.

*N-(3-(trifluoromethyl)-1-methyl-1H-pyrazol-5-yl)-2-(3-fluorophenyl)acetamide* (**C-9**)

Yellow solid, yield 76%; mp = 131.6**–**133.8 ℃; ^1^H NMR (400 MHz, DMSO-*d*_6_) δ 10.50 (s, 1H), 7.39 (q, *J* = 8.0 Hz, 1H), 7.18 (d, *J* = 7.8 Hz, 2H), 7.11 (t, *J* = 8.8 Hz, 1H), 6.66 (s, 1H), 3.79 (d, *J* = 7.4 Hz, 5H); ^13^C NMR (101 MHz, DMSO-*d*_6_) δ 169.3, 162.5 (d, *J* = 243.1 Hz), 139.3 (q, *J* = 35.7 Hz), 138.4, 138.3, 130.6 (d, *J* = 8.5 Hz), 125.9, 121.8 (q, *J* = 804.1 Hz), 116.6 (d, *J* = 21.5 Hz), 114.0 (d, *J* = 20.9 Hz), 97.4, 41.9, 36.9; ESI-MS calculated for C_13_H_11_F_4_N_4_ONa [M+Na]^+^, 324.0731; found 324.0728.

*N-(3-(trifluoromethyl)-1-methyl-1H-pyrazol-5-yl)-2-(4-fluorophenyl)acetamide* (**C-10**)

Yellow solid, yield 74%; mp = 132.6**–**134.5 ℃; ^1^H NMR (400 MHz, DMSO-*d*_6_) δ 10.46 (s, 1H), 7.38 (dd, *J* = 8.3, 5.8 Hz, 2H), 7.17 (t, *J* = 8.9 Hz, 2H), 6.65 (s, 1H), 3.76 (d, *J* = 6.6 Hz, 5H); ^13^C NMR (101 MHz, DMSO-*d*_6_) δ 169.7, 161.7 (d, *J* = 242.4 Hz), 139.2 (q, *J* = 36.9 Hz), 138.5, 131.8 (d, *J* = 3.0 Hz), 131.6 (d, *J* = 8.1 Hz), 125.8, 121.8 (q, *J* = 268.1 Hz), 115.5 (d, *J* = 21.2 Hz), 97.3, 41.5, 36.9; ESI-MS calculated for C_13_H_11_F_4_N_4_ONa [M+Na]^+^, 324.0731; found 324.0725.

*2-(2-chlorophenyl)-N-(3-(trifluoromethyl)-1-methyl-1H-pyrazol-5-yl)acetamide* (**C-11**)

Yellow solid, yield 86%; mp = 163.5**–**165.1 ℃; ^1^H NMR (400 MHz, Chloroform-*d*) δ 7.45 (dt, *J* = 24.6, 4.5 Hz, 2H), 7.38–7.31 (m, 2H), 7.13 (s, 1H), 6.47 (s, 1H), 3.90 (s, 2H), 3.70 (s, 3H); ^13^C NMR (101 MHz, Chloroform-*d*) δ 168.1, 135.8, 134.2, 134.0, 131.9, 131.8, 130.2, 129.9, 127.9, 122.2, 99.1, 41.6, 36.3; ESI-MS calculated for C_13_H_11_ClF_3_N_3_ONa [M+Na]^+^, 340.0435; found 340.0435.

*2-(3-chlorophenyl)-N-(3-(trifluoromethyl)-1-methyl-1H-pyrazol-5-yl)acetamide* (**C-12**)

White solid, yield 82%; mp = 127.1**–**130.9 ℃; ^1^H NMR (400 MHz, DMSO-*d*_6_) δ 10.48 (s, 1H), 7.42–7.34 (m, 4H), 6.65 (s, 1H), 3.77 (s, 5H); ^13^C NMR (101 MHz, DMSO-*d*_6_) δ 169.3, 139.2 (q, *J* = 38.1, 37.7 Hz), 138.5, 138.0, 133.3, 130.6, 129.7, 128.5, 127.2, 121.8 (q, *J* = 268.3 Hz), 97.3, 41.8, 36.9; ESI-MS calculated for C_13_H_11_ClF_3_N_3_ONa [M+Na]^+^, 340.0435; found 340.0435.

*2-(4-chlorophenyl)-N-(3-(trifluoromethyl)-1-methyl-1H-pyrazol-5-yl)acetamide* (**C-13**)

Yellow solid, yield 83%; mp = 157.3**–**159.1 ℃; ^1^H NMR (400 MHz, DMSO-*d*_6_) δ 10.5 (s, 1H), 7.4 (q, *J* = 8.5 Hz, 4H), 6.7 (s, 1H), 3.8 (s, 5H); ^13^C NMR (101 MHz, DMSO-*d*_6_) δ 169.4, 139.2 (q, *J* = 38.3, 37.5 Hz), 138.5, 134.6, 131.9, 131.6, 128.7, 121.8 (q, *J* = 268.1 Hz), 97.4, 41.6, 36.9; ESI-MS calculated for C_13_H_11_ClF_3_N_3_ONa [M+Na]^+^, 340.0435; found 340.0434.

*N-(3-(trifluoromethyl)-1-methyl-1H-pyrazol-5-yl)-2-(2-(trifluoromethyl)phenyl)acetamide* (**C-14**)

Yellow solid, yield 77%; mp = 173.3**–**174.9 ℃; ^1^H NMR (400 MHz, DMSO-*d*_6_) δ 10.5 (s, 1H), 7.7 (d, *J* = 7.8 Hz, 1H), 7.7 (t, *J* = 7.5 Hz, 1H), 7.5 (dd, *J* = 18.2, 7.9 Hz, 2H), 6.6 (s, 1H), 4.0 (s, 2H), 3.8 (s, 3H); ^13^C NMR (101 MHz, DMSO-*d*_6_) δ 168.9, 139.3 (q, *J* = 37.4 Hz), 138.5, 134.0, 133.6, 133.6, 132.8, 128.0 (q, *J* = 29.3 Hz), 126.2 (q, *J* = 5.5 Hz), 126.1, 126.1, 125.8, 123.3 (d, *J* = 48.4 Hz), 120.4, 97.4, 36.9; ESI-MS calculated for C_14_H_11_F_6_N_3_ONa [M+Na]^+^, 374.0699; found 374.0697.

*N-(3-(trifluoromethyl)-1-methyl-1H-pyrazol-5-yl)-2-(3-(trifluoromethyl)phenyl)acetamide* (**C-15**)

White solid, yield 75%; mp = 166.8**–**170.1 ℃; ^1^H NMR (400 MHz, DMSO-*d*_6_) δ 10.56 (s, 1H), 7.72 (s, 1H), 7.65 (d, *J* = 7.6 Hz, 2H), 7.62–7.56 (m, 1H), 6.67 (s, 1H), 3.91 (s, 2H), 3.79 (s, 3H); ^13^C NMR (101 MHz, DMSO-*d*_6_) δ 169.2, δ 139.2 (q, *J* = 37.6 Hz), 138.5, 137.0, 134.0, 129.8, 129.5 (q, *J* = 30.9 Hz), 126.5 (q, *J* = 4.1 Hz), 126.1, 124.0, 123.2 (q, *J* = 25.7 Hz), 120.4, 97.3, 41.8, 36.9; ESI-MS calculated for C_14_H_11_F_6_N_3_ONa [M+Na]^+^, 374.0699; found 374.0697.

*N-(3-(trifluoromethyl)-1-methyl-1H-pyrazol-5-yl)-2-(4-(trifluoromethyl)phenyl)acetamide* (**C-16**)

Yellow solid, yield 71%; mp = 138.1**–**141.1 ℃; ^1^H NMR (400 MHz, DMSO-*d*_6_) δ 10.55 (s, 1H), 7.71 (d, *J* = 7.2 Hz, 2H), 7.57 (d, *J* = 7.2 Hz, 2H), 6.66 (s, 1H), 3.89 (s, 2H), 3.78 (s, 3H); ^13^C NMR (101 MHz, DMSO-*d*_6_) δ 169.2, 139.3 (q, *J* = 37.7 Hz), 138.5, 137.0, 134.0, 129.8, 129.5 (q, *J* = 31.2 Hz), 126.5 (q, *J* = 3.7 Hz), 126.1, 124.0, 123.2 (q, *J* = 25.7 Hz), 97.3, 41.8, 36.9; ESI-MS calculated for C_14_H_11_F_6_N_3_ONa [M+Na]^+^, 374.0699; found 374.0697.

*N-(3-(difluoromethyl)-1-methyl-1H-pyrazol-5-yl)-2-phenylacetamide* (**C-17**)

Yellow solid, yield 82%; mp =92.6**–**93.0 ℃; ^1^H NMR (400 MHz, DMSO-*d*_6_) δ 10.31 (s, 1H), 7.29 (d, *J* = 5.0 Hz, 4H), 7.24–7.18 (m, 1H), 6.42 (s, 1H), 3.68 (s, 2H), 3.65 (s, 3H); ^13^C NMR (101 MHz, DMSO-*d*_6_) δ 169.7, 144.3 (t, *J* = 28.6 Hz), 138.1, 135.8, 129.6, 128.8, 127.2, 112.0 (t, *J* = 231.5 Hz), 96.5, 42.5, 36.5; ESI-MS calculated for C_13_H_13_F_2_N_3_ONa [M+Na]^+^, 288.0919; found 288.0920.

*N-(3-(difluoromethyl)-1-methyl-1H-pyrazol-5-yl)-2-(o-tolyl)acetamide* (**C-18**)

Yellow solid, yield 85%; mp = 164.5**–**165.9 ℃; ^1^H NMR (400 MHz, DMSO-*d*_6_) δ 10.35 (s, 1H), 7.29–7.22 (m, 1H), 7.22–7.12 (m, 3H), 6.48 (s, 1H), 3.77 (s, 2H), 3.73 (s, 3H), 2.30 (s, 3H); ^13^C NMR (101 MHz, DMSO-*d*_6_) δ 169.7, 144.30 (t, *J* = 28.4 Hz), 138.2, 137.2, 134.5, 130.5, 127.3, 126.3, 112.01 (t, *J* = 231.6 Hz), 96.5, 36.5, 19.8; ESI-MS calculated for C_14_H_15_F_2_N_3_ONa [M+Na]^+^, 302.1075; found 302.1070.

*N-(3-(difluoromethyl)-1-methyl-1H-pyrazol-5-yl)-2-(m-tolyl)acetamide* (**C-19**)

White solid, yield 66%; mp = 122.5**–**124.6 ℃; ^1^H NMR (400 MHz, Chloroform-*d*) δ 7.32 (t, *J* = 7.5 Hz, 1H), 7.21–7.11 (m, 3H), 6.94 (s, 1H), 6.40 (s, 1H), 3.74 (s, 2H), 3.59 (s, 3H), 2.39 (s, 3H); ^13^C NMR (101 MHz, Chloroform-*d*) δ 169.5, 145.2 (t, *J* = 29.4 Hz), 139.4, 135.9, 133.6, 130.2, 129.4, 128.9, 126.4, 111.1 (t, *J* = 234.2 Hz), 97.8, 43.7, 35.8, 21.4; ESI-MS calculated for C_14_H_15_F_2_N_3_ONa [M+Na]^+^, 302.1075; found 302.1069.

*N-(3-(difluoromethyl)-1-methyl-1H-pyrazol-5-yl)-2-(p-tolyl)acetamide* (**C-20**)

Yellow solid, yield 71%; mp = 160.1**–**162.0 ℃; ^1^H NMR (400 MHz, DMSO-*d*_6_) δ 10.35 (s, 1H), 7.26 (d, *J* = 6.5 Hz, 2H), 7.18 (d, *J* = 6.7 Hz, 2H), 6.49 (s, 1H), 3.72 (d, *J* = 10.3 Hz, 5H), 2.32 (s, 3H); ^13^C NMR (101 MHz, DMSO-*d*_6_) δ 169.9, 144.3 (t, *J* = 28.5 Hz), 138.2, 136.2, 132.7, 129.4, 112.0 (t, *J* = 231.5 Hz), 96.5, 42.1, 36.5, 21.1; ESI-MS calculated for C_14_H_15_F_2_N_3_ONa [M+Na]^+^, 302.1075; found 302.1072.

*N-(3-(difluoromethyl)-1-methyl-1H-pyrazol-5-yl)-2-(2-methoxyphenyl)acetamide* (**C-21**)

Yellow solid, yield 87%; mp = 103.5**–**105.3 ℃; ^1^H NMR (400 MHz, DMSO-*d*_6_) δ 10.17 (s, 1H), 7.19 (q, *J* = 7.8 Hz, 2H), 6.93 (d, *J* = 8.1 Hz, 1H), 6.85 (d, *J* = 14.8 Hz, 1H), 6.38 (s, 1H), 3.72 (s, 3H), 3.67 (s, 3H), 3.63 (s, 2H); ^13^C NMR (101 MHz, DMSO-*d*_6_) δ 169.9, 157.7, 144.3 (t, *J* = 28.9 Hz), 138.3, 131.5, 128.7, 124.1, 120.6, 112.0 (t, *J* = 231.5 Hz), 111.1, 96.6, 55.8, 37.4, 36.5; ESI-MS calculated for C_14_H_15_F_2_N_3_O_2_Na [M+Na]^+^, 318.1025; found 318.1023.

*N-(3-(difluoromethyl)-1-methyl-1H-pyrazol-5-yl)-2-(3-methoxyphenyl)acetamide* (**C-22**)

White solid, yield 65%; mp = 100.1**–**103.2 ℃; ^1^H NMR (400 MHz, DMSO-*d*_6_) δ 10.32 (s, 1H), 7.25 (t, *J* = 8.0 Hz, 1H), 6.92 (s, 2H), 6.90–6.82 (m, 2H), 6.45 (s, 1H), 3.75 (s, 3H), 3.70 (d, *J* = 3.3 Hz, 5H); ^13^C NMR (101 MHz, DMSO-*d*_6_) δ 169.6, 159.7, 144.3 (t, *J* = 28.4 Hz), 138.1, 137.2, 129.8, 121.8, 115.4, 112.6, 112.0 (t, *J* = 231.6 Hz), 96.6, 55.4, 42.6, 36.5; ESI-MS calculated for C_14_H_15_F_2_N_3_O_2_Na [M+Na]^+^, 318.1025; found 318.1022.

*N-(3-(difluoromethyl)-1-methyl-1H-pyrazol-5-yl)-2-(4-methoxyphenyl)acetamide* (**C-23**)

Yellow solid, yield 75%; mp = 99.9**–**102.4 ℃; ^1^H NMR (400 MHz, DMSO-*d*_6_) δ 10.31 (s, 1H), 7.26 (d, *J* = 8.5 Hz, 2H), 6.91 (d, *J* = 8.5 Hz, 2H), 3.74 (s, 3H), 3.70 (s, 3H), 3.65 (s, 2H); ^13^C NMR (101 MHz, DMSO-*d*_6_) δ 170.1, 158.6, 144.3 (t, *J* = 28.7 Hz), 138.2, 130.6, 127.7, 114.3, 112.0 (t, *J* = 231.5 Hz), 96.5, 55.5, 41.6, 36.5; ESI-MS calculated for C_14_H_15_F_2_N_3_O_2_Na [M+Na]^+^, 318.1025; found 318.1022.

*N-(3-(difluoromethyl)-1-methyl-1H-pyrazol-5-yl)-2-(2-fluorophenyl)acetamide* (**C-24**)

Yellow solid, yield 82%; mp =119.5**–**122.5 ℃; ^1^H NMR (400 MHz, DMSO-*d*_6_) δ 10.40 (s, 1H), 7.40 (t, *J* = 7.6 Hz, 1H), 7.33 (q, *J* = 6.6, 5.7 Hz, 1H), 7.19 (q, *J* = 7.2 Hz, 2H), 6.45 (s, 1H), 3.82 (s, 2H), 3.73 (s, 3H); ^13^C NMR (101 MHz, DMSO-*d*_6_) δ 168.8, 161.1 (d, *J* = 244.3 Hz), 144.3 (t, *J* = 28.4 Hz), 138.1, 132.5 (d, *J* = 4.4 Hz), 129.5 (d, *J* = 8.1 Hz), 124.7 (d, *J* = 3.4 Hz), 122.9 (d, *J* = 15.9 Hz), 115.5 (d, *J* = 21.5 Hz), 112.0 (t, *J* = 231.6 Hz), 96.5, 36.5, 35.8; ESI-MS calculated for C_13_H_12_F_3_N_3_ONa [M+Na]^+^, 306.0825; found 306.0820.

*N-(3-(difluoromethyl)-1-methyl-1H-pyrazol-5-yl)-2-(3-fluorophenyl)acetamide* (**C-25**)

White solid, yield 84%; mp = 110.8**–**112.2 ℃; ^1^H NMR (400 MHz, DMSO-*d*_6_) δ 10.37 (s, 1H), 7.38 (q, *J* = 7.9 Hz, 1H), 7.18 (d, *J* = 8.5 Hz, 2H), 7.10 (t, *J* = 9.2 Hz, 1H), 6.46 (s, 1H), 3.77 (s, 2H), 3.71 (s, 3H); ^13^C NMR (101 MHz, DMSO-*d*_6_) δ 169.2, 162.5 (d, *J* = 243.1 Hz), 144.3 (t, *J* = 28.4 Hz), 138.5 (d, *J* = 8.0 Hz), 138.1, 130.6 (d, *J* = 8.4 Hz), 125.9 (d, *J* = 2.7 Hz), 16.5 (d, *J* = 21.5 Hz), 114.0 (d, *J* = 20.8 Hz), 112.0 (t, *J* = 231.6 Hz), 96.5, 42.0, 36.5; ESI-MS calculated for C_13_H_12_F_3_N_3_ONa [M+Na]^+^, 306.0825; found 306.0821.

*N-(3-(difluoromethyl)-1-methyl-1H-pyrazol-5-yl)-2-(4-fluorophenyl)acetamide* (**C-26**)

Yellow solid, yield 72%; mp = 129.9**–**131.0 ℃; ^1^H NMR (400 MHz, DMSO-*d*_6_) δ 10.36 (s, 1H), 7.41–7.34 (m, 2H), 7.17 (t, *J* = 8.9 Hz, 2H), 6.47 (s, 1H), 3.74 (s, 2H), 3.71 (s, 3H); ^13^C NMR (101 MHz, DMSO-*d*_6_) δ 169.6, 161.6 (d, *J* = 242.4 Hz), 144.3 (t, *J* = 28.4 Hz), 138.1, 131.93 (d, *J* = 3.1 Hz), 131.6 (d, *J* = 8.1 Hz), 115.5 (d, *J* = 21.2 Hz), 115.4, 111.2 (t, *J* = 231.5 Hz), 96.5, 41.5, 36.5; ESI-MS calculated for C_13_H_12_F_3_N_3_ONa [M+Na]^+^, 306.0825; found 306.0820.

*2-(2-chlorophenyl)-N-(3-(difluoromethyl)-1-methyl-1H-pyrazol-5-yl)acetamide* (**C-27**)

Yellow solid, yield 69%; mp = 146.5**–**148.1 ℃; ^1^H NMR (400 MHz, DMSO-*d*_6_) δ 10.17 (s, 1H), 7.19 (q, *J* = 7.8 Hz, 2H), 6.93 (d, *J* = 8.1 Hz, 1H), 6.85 (d, *J* = 14.8 Hz, 1H), 6.38 (s, 1H), 3.72 (s, 3H), 3.67 (s, 3H), 3.63 (s, 2H); ^13^C NMR (101 MHz, DMSO-*d*_6_) δ 168.7, 144.3 (t, *J* = 28.3 Hz), 138.1, 134.2, 133.9, 132.8, 129.5, 129.3, 127.6, 112.0 (t, *J* = 231.5 Hz), 96.5, 36.6; ESI-MS calculated for C_13_H_12_ClF_2_N_3_ONa [M+Na]^+^, 322.0529; found 322.0531.

*2-(3-chlorophenyl)-N-(3-(difluoromethyl)-1-methyl-1H-pyrazol-5-yl)acetamide* (**C-28**)

White solid, yield 77%; mp = 134.9**–**137.1 ℃; ^1^H NMR (400 MHz, DMSO-*d*_6_) δ 10.37 (s, 1H), 7.42 (s, 1H), 7.40–7.34 (m, 2H), 7.30 (d, *J* = 7.2 Hz, 1H), 6.87 (s, 1H), 6.46 (s, 1H), 3.77 (s, 2H), 3.72 (s, 3H); ^13^C NMR (101 MHz, DMSO-*d*_6_) δ 169.2, 144.3 (t, *J* = 28.4 Hz), 138.2, 133.3, 130.6, 129.7, 128.5, 127.2, 112.0 (t, *J* = 231.6 Hz), 96.5, 41.8, 36.5; ESI-MS calculated for C_13_H_12_ClF_2_N_3_ONa [M+Na]^+^, 322.0529; found 322.0530.

*2-(4-chlorophenyl)-N-(3-(difluoromethyl)-1-methyl-1H-pyrazol-5-yl)acetamide* (**C-29**)

Yellow solid, yield 75%; mp = 146.5**–**148.1 ℃; ^1^H NMR (400 MHz, DMSO-*d*_6_) δ 10.29 (s, 1H), 7.31 (d, *J* = 9.5 Hz, 4H), δ 6.80 (t, *J* = 54.6 Hz, 1H), 6.38 (s, 1H), 3.67 (s, 2H), 3.64 (s, 3H); ^13^C NMR (101 MHz, DMSO-*d*_6_) δ 169.4, 144.3 (t, *J* = 28.6 Hz), 138.1, 134.8, 131.9, 131.6, 128.7, 112.0 (t, *J* = 231.5 Hz), 96.5, 41.7, 36.5; ESI-MS calculated for C_13_H_12_ClF_2_N_3_ONa [M+Na]^+^, 322.0529; found 322.0530.

*N-(3-(difluoromethyl)-1-methyl-1H-pyrazol-5-yl)-2-(2-(trifluoromethyl)phenyl)acetamide* (**C-30**)

Yellow solid, yield 66%; mp = 171.4**–**173.4 ℃;^1^H NMR (400 MHz, DMSO-*d*_6_) δ 10.40 (s, 1H), 7.73 (s, 1H), 7.66 (d, *J* = 15.0 Hz, 1H), 7.52 (dd, *J* = 18.9, 7.7 Hz, 2H), 6.43 (s, 1H), 4.01 (s, 2H), 3.73 (s, 3H); ^13^C NMR (101 MHz, DMSO-*d*_6_) δ 168.8, 144.3 (t, *J* = 28.4 Hz), 138.1, 134.0, 133.7, 133.7, 132.8, 128.0 (q, *J* = 29.4 Hz), 127.9, 126.3, 126.2 (q, *J* = 5.5 Hz), 123.6, 112.0 (t, *J* = 231.6 Hz), 39.2, 36.5; ESI-MS calculated for C_14_H_12_F_5_N_3_ONa [M+Na]^+^, 356.0793; found 356.0792.

*N-(3-(difluoromethyl)-1-methyl-1H-pyrazol-5-yl)-2-(3-(trifluoromethyl)phenyl)acetamide* (**C-31**)

White solid, yield 63%; mp = 145.2**–**146.3 ℃; ^1^H NMR (400 MHz, Chloroform-*d*) δ 7.32 (t, *J* = 7.5 Hz, 1H), 7.21–7.11 (m, 3H), 6.94 (s, 1H), 6.40 (s, 1H), 3.74 (s, 2H), 3.59 (s, 3H), 2.39 (s, 3H); ^13^C NMR (101 MHz, DMSO-*d*_6_) δ 169.2, 139.2 (q, *J* = 37.6 Hz), 138.5, 137.0, 134.0, 129.8, 129.5 (q, *J* = 30.9 Hz), 126.5 (q, *J* = 3.7 Hz), 126.1, 124.0, 124.0, 123.2 (q, *J* = 25.7 Hz), 120.4, 97.3, 41.8, 36.9; ESI-MS calculated for C_14_H_12_F_5_N_3_ONa [M+Na]^+^, 356.0793; found 356.0793.

*N-(3-(difluoromethyl)-1-methyl-1H-pyrazol-5-yl)-2-(4-(trifluoromethyl)phenyl)acetamide* (**C-32**)

Yellow solid, yield 67%; mp = 136.4**–**138.0 ℃; ^1^H NMR (400 MHz, DMSO-*d*_6_) δ 10.45 (s, 1H), 7.71 (d, *J* = 8.0 Hz, 2H), 7.57 (d, *J* = 7.9 Hz, 2H), 6.47 (d, *J* = 3.8 Hz, 1H), 3.87 (s, 2H), 3.73 (s, 3H); ^13^C NMR (101 MHz, DMSO-*d*_6_) δ 169.0, 144.3 (t, *J* = 28.4 Hz), 140.6, 138.0, 130.6, 127.9 (q, *J* = 32.0 Hz), 125.6, 124.8 (d, *J* = 271.9 Hz), 112.0 (t, *J* = 231.5 Hz), 96.5, 42.1, 36.5; ESI-MS calculated for C_14_H_12_F_5_N_3_ONa [M+Na]^+^, 356.0793; found 356.0791.

*2-(2,4-dichlorophenyl)-N-(1-methyl-3-(trifluoromethyl)-1H-pyrazol-5-yl)acetamide* (**C-33**)

White solid, yield 75%; mp = 192.1**–**195.3 ℃; ^1^H NMR (400 MHz, DMSO-*d*_6_) δ 10.55 (s, 1H), 7.63 (s, 1H), 7.48 (d, *J* = 8.3 Hz, 1H), 7.43 (d, *J* = 9.6 Hz, 1H), 6.63 (s, 1H), 3.93 (s, 2H), 3.79 (s, 3H); ^13^C NMR (101 MHz, DMSO-*d*_6_) δ 168.4, 139.2 (q, *J* = 37.4 Hz), 138.5, 135.2, 134.1, 133.0, 132.9, 129.0, 127.7, 121.8 (q, *J* = 268.0 Hz), 97.4, 37.0; ESI-MS calculated for C_13_H_11_Cl_2_F_3_N_3_O [M+H]^+^, 352.0226; found 352.0226.

*2-(3,4-dichlorophenyl)-N-(1-methyl-3-(trifluoromethyl)-1H-pyrazol-5-yl)acetamide* (**C-34**)

White solid, yield 72%; mp = 197.6**–**198.9 ℃; ^1^H NMR (400 MHz, DMSO-*d*_6_) δ 10.49 (s, 1H), 7.62 (s, 2H), 7.32 (s, 1H), 6.65 (s, 1H), 3.80 (s, 2H), 3.77 (s, 3H); ^13^C NMR (101 MHz, DMSO-*d*_6_) δ 169.0, 139.2 (q, *J* = 37.3 Hz), 138.4, 136.7, 131.9, 131.2, 130.9, 130.3, 129.9, 121.8 (q, *J* = 268.0 Hz), 97.3, 41.1, 37.0; ESI-MS calculated for C_13_H_11_Cl_2_F_3_N_3_O [M+H]^+^, 352.0226; found 352.0226.

*2-(3,5-dichlorophenyl)-N-(1-methyl-3-(trifluoromethyl)-1H-pyrazol-5-yl)acetamide* (**C-35**)

White solid, yield 79%; mp = 190.2**–**193.3 ℃; ^1^H NMR (400 MHz, DMSO-*d*_6_) δ 10.51 (s, 1H), 7.53 (s, 1H), 7.41 (s, 2H), 6.66 (s, 1H), 3.82 (s, 2H), 3.78 (s, 3H); ^13^C NMR (101 MHz, DMSO-*d*_6_) δ 168.8, 139.8, 139.7, 139.2 (q, *J* = 37.4 Hz), 138.7, 138.4, 134.2, 128.8, 126.9, 125.8, 121.8 (q, *J* = 268.0 Hz), 117.8, 97.3, 41.3, 37.0; ESI-MS calculated for C_13_H_11_Cl_2_F_3_N_3_O [M+H]^+^, 352.0226; found 352.0222.

*2-(2,4-bis(trifluoromethyl)phenyl)-N-(1-methyl-3-(trifluoromethyl)-1H-pyrazol-5-yl)acetamide* (**C-36**)

White solid, yield 61%; mp = 168.2**–**170.1 ℃; ^1^H NMR (400 MHz, DMSO-*d*_6_) δ 10.62 (s, 1H), 8.10 (s, 1H), 8.01 (s, 1H), 7.83 (s, 1H), 6.62 (s, 1H), 4.15 (s, 2H), 3.79 (s, 3H).^13^C NMR (101 MHz, DMSO-*d*_6_) δ 168.2, 139.4 (q, *J* = 37.4 Hz), 138.7, 135.5, 129.5, 129.3, 128.8 (q, *J* = 31.7 Hz), 124.0 (q, *J* = 274.1 Hz), 123.9 (q, *J* = 272.3 Hz), 123.0, 121.7 (q, *J* = 267.9 Hz), 117.7, 97.3, 39.1, 36.8; ESI-MS calculated for C_15_H_11_F_9_N_3_O [M+H]^+^, 420.0753; found 420.0754.

*2-(3,5-bis(trifluoromethyl)phenyl)-N-(1-methyl-3-(trifluoromethyl)-1H-pyrazol-5-yl)acetamide* (**C-37**)

White solid, yield 65%; mp = 186.0**–**188.4 ℃; ^1^H NMR (400 MHz, DMSO-*d*_6_) δ 10.60 (s, 1H), 8.06 (s, 2H), 8.00 (s, 1H), 6.65 (s, 1H), 4.06 (s, 2H), 3.80 (s, 3H).^13^C NMR (101 MHz, DMSO-*d*_6_) δ 168.8, 139.3 (q, *J* = 37.4 Hz), 138.9, 138.4, 131.1（2C）, 130.5 (q, *J* = 32.7 Hz), 123.8 (q, *J* = 272.7 Hz)，121.8 (q, *J* = 268.1 Hz)，121.0, 121.0, 120.9，97.3, 41.2, 36.9; ESI-MS calculated for C_15_H_11_F_9_N_3_O [M+H]^+^, 420.0753; found 420.0754.

*N-(1-methyl-3-(trifluoromethyl)-1H-pyrazol-5-yl)-2-(naphthalen-2-yl)acetamide* (**C-38**)

White solid, yield 65%; mp = 186.4**–**187.7 ℃; ^1^H NMR (400 MHz, DMSO-*d*_6_) δ 10.55 (s, 1H), 7.89 (d, *J* = 8.0 Hz, 3H), 7.85 (s, 1H), 7.51 (d, *J* = 6.5 Hz, 3H), 6.66 (s, 1H), 3.93 (s, 2H), 3.78 (s, 3H); ^13^C NMR (101 MHz, DMSO-*d*_6_) δ 169.8, 139.2 (q, *J* = 37.5 Hz), 138.6, 133.4, 133.3, 132.4, 128.3, 128.1, 128.1, 128.0, 127.9, 126.6, 126.2, 121.8 (q, *J* = 268.2 Hz), 97.4, 42.6, 37.0; ESI-MS calculated for C_17_H_15_F_3_N_3_O [M+H]^+^, 334.1162; found 334.1162.

*N-(4-chloro-1-methyl-3-(trifluoromethyl)-1H-pyrazol-5-yl)-2-(3-chlorophenyl)acetamide* (**C-39**)

White solid, yield 81%; mp = 167.1**–**170.2 ℃; ^1^H NMR (400 MHz, DMSO-*d*_6_) δ 10.77 (s, 1H), 7.45 (s, 1H), 7.33 (s, 3H), 3.81 (s, 2H), 3.70 (s, 3H); ^13^C NMR (101 MHz, DMSO-*d*_6_) δ 170.4, 137.9, 136.1, 135.4 (q, *J* = 36.6 Hz), 133.4, 130.7, 129.6, 128.4, 127.3, 120.9 (q, *J* = 268.8 Hz), 103.2, 41.5, 37.8; ESI-MS calculated for C_13_H_9_Cl_2_F_3_N_3_O [M-H]^-^, 350.0080; found 350.0070.

*N-(4-chloro-1-methyl-3-(trifluoromethyl)-1H-pyrazol-5-yl)-2-(4-chlorophenyl)acetamide* (**C-40**)

Yellow solid, yield 79%; mp = 181.3**–**183.0 ℃; ^1^H NMR (400 MHz, DMSO-*d*_6_) δ 10.63 (s, 1H), 7.44–7.36 (m, 4H), 3.78 (s, 2H), 3.69 (s, 3H);^13^C NMR (101 MHz, DMSO-*d*_6_) δ 170.6, 136.1, 135.4 (q, *J* = 37.0 Hz), 134.5, 132.0, 131.6, 128.8, 120.9 (q, *J* = 268.5 Hz), 103.2, 41.3, 37.8; ESI-MS calculated for C_13_H_9_Cl_2_F_3_N_3_O [M-H]^-^, 350.0080; found 350.0070.

*2-(3-chlorophenyl)-N-(4-fluoro-1-methyl-3-(trifluoromethyl)-1H-pyrazol-5-yl)acetamide* (**C-41**)

Yellow solid, yield 82%; mp = 119.6**–**121.0 ℃; ^1^H NMR (400 MHz, DMSO-*d*_6_) δ 10.43 (s, 1H), 7.36 (ddd, *J* = 28.2, 16.5, 8.4 Hz, 4H), 3.80 (s, 2H), 3.68 (s, 3H); ^13^C NMR (101 MHz, DMSO-*d*_6_) δ 170.3, 138.6 (d, *J* = 251.6 Hz), 137.8, 133.4, 130.7, 129.7, 128.5, 127.3, 125.4 (qd, *J* = 38.2, 5.7 Hz), 124.8 (d, *J* = 22.3 Hz), 120.7 (qd, *J* = 267.8, 3.4 Hz), 41.4, 37.7; ESI-MS calculated for C_13_H_9_ClF_4_N_3_O [M-H]^-^, 334.0376; found 334.0368.

*2-(4-chlorophenyl)-N-(4-fluoro-1-methyl-3-(trifluoromethyl)-1H-pyrazol-5-yl)acetamide* (**C-42**)

Yellow solid, yield 85%; mp = 133.5**–**136.2 ℃; ^1^H NMR (400 MHz, DMSO-*d*_6_) δ 10.51 (s, 1H), 7.40 (s, 4H), 3.78 (s, 2H), 3.67 (s, 3H). ^13^C NMR (101 MHz, DMSO-*d*_6_) δ 170.5, 138.5 (d, *J* = 252.2 Hz), 134.4, 132.0, 131.6, 128.8, 125.4 (qd, *J* = 38.4, 6.4 Hz), 124.8 (d, *J* = 22.4 Hz), 120.7 (qd, *J* = 267.8, 3.5 Hz), 41.2, 37.7; ESI-MS calculated for C_13_H_9_ClF_4_N_3_O [M-H]^-^, 334.0376; found 334.0368.

*N-(4-chloro-1-methyl-3-(trifluoromethyl)-1H-pyrazol-5-yl)-2-(3(trifluoromethyl)phenyl)acetamide* (**C-43**)

White solid, yield 65%; mp = 161.2**–**163.3 ℃; ^1^H NMR (400 MHz, DMSO-*d*_6_) δ 10.56 (s, 1H), 7.74 (s, 1H), 7.69–7.57 (m, 3H), 3.92 (s, 2H), 3.71 (s, 3H); ^13^C NMR (101 MHz, DMSO-*d*_6_) δ 170.4, 136.8, 136.0, 135.4 (q, *J* = 37.2 Hz), 134.0, 129.9, 129.7, 129.4, 126.3 (q, *J* = 3.9 Hz), 124.1 (q, *J* = 3.9 Hz), 122.8 (q, *J* = 110.5 Hz), 103.2, 41.5, 37.8; ESI-MS calculated for C_14_H_9_ClF_6_N_3_O [M-H]^-^, 384.0344; found 384.0335.

*N-(4-chloro-1-methyl-3-(trifluoromethyl)-1H-pyrazol-5-yl)-2-(4(trifluoromethyl)phenyl)acetamide* (**C-44**)

Yellow solid, yield 62%; mp = 174.2**–**175.5 ℃; ^1^H NMR (400 MHz, DMSO-*d*_6_) δ 10.57 (s, 1H), 7.72 (d, *J* = 8.1 Hz, 2H), 7.59 (s, 2H), 3.90 (s, 2H), 3.70 (s, 3H); ^13^C NMR (101 MHz, DMSO-*d*_6_) δ 170.3, 145.8, 140.2, 136.0, 135.4 (q, *J* = 37.3 Hz), 130.6, 128.0 (q, *J* = 31.7 Hz), 125.7 (q, *J* = 3.8 Hz), 125.6, 124.9, 122.8 (q, *J* = 120.7 Hz), 119.6, 103.2, 41.7, 37.8; ESI-MS calculated for C_14_H_9_ClF_6_N_3_O [M-H]^-^, 384.0344; found 384.0334.

*N-(4-fluoro-1-methyl-3-(trifluoromethyl)-1H-pyrazol-5-yl)-2-(3-(trifluoromethyl)phenyl)acetamide* (**C-45**)

Yellow solid, yield 71%; mp = 172.5**–**173.6 ℃; ^1^H NMR (400 MHz, DMSO-*d*_6_) δ 10.47 (s, 1H), 7.73 (s, 1H), 7.65 (d, *J* = 7.7 Hz, 2H), 7.59 (dd, *J* = 8.7, 6.4 Hz, 1H), 3.91 (s, 2H), 3.69 (s, 3H); ^13^C NMR (101 MHz, DMSO-*d*_6_) δ 170.4, 138.6 (d, *J* = 253.0 Hz), 136.7, 134.0, 129.8, 129.5 (q, *J* = 31.5 Hz), 126.4 (q, *J* = 3.8 Hz), 126.0, 124.8 (q, *J* = 22.3 Hz), 124.0 (q, *J* = 3.7 Hz), 122.1 (d, *J* = 3.5 Hz), 119.4 (d, *J* = 3.4 Hz), 41.4, 37.7; ESI-MS calculated for C_14_H_9_F_7_N_3_O [M-H]^-^, 368.0639; found 368.0628.

*N-(4-fluoro-1-methyl-3-(trifluoromethyl)-1H-pyrazol-5-yl)-2-(4-(trifluoromethyl)phenyl)acetamide* (**C-46**)

White solid, yield 66%; mp = 169.2**–**172.1 ℃; ^1^H NMR (400 MHz, DMSO-*d*_6_) δ 10.50 (s, 1H), 7.72 (d, *J* = 8.0 Hz, 2H), 7.59 (s, 2H), 3.90 (s, 2H), 3.69 (s, 3H); ^13^C NMR (101 MHz, DMSO-*d*_6_) δ 170.2, 140.2, 138.6 (d, *J* = 252.7 Hz), 130.6, 128.0 (q, *J* = 31.9 Hz), 125.7, 124.8 (q, *J* = 22.3 Hz), 120.7 (qd, *J* = 268.0, 3.1 Hz), 41.7, 37.7; ESI-MS calculated for C_14_H_9_F_7_N_3_O [M-H]^-^, 368.0639; found 368.0625.

*N-(4-chloro-1-methyl-3-(trifluoromethyl)-1H-pyrazol-5-yl)-2-(m-tolyl)acetamide* (**C-47**)

Yellow solid, yield 81%; mp = 106.2**–**108.5 ℃; ^1^H NMR (400 MHz, Chloroform-*d*) δ 7.32 (t, *J* = 7.8 Hz, 1H), 7.17 (t, *J* = 8.7 Hz, 3H), 6.88 (s, 1H), 3.77 (s, 2H), 3.72 (s, 3H), 2.38 (s, 3H); ^13^C NMR (101 MHz, Chloroform-*d*) δ 170.6, 139.5, 133.5 (q, *J* = 41.9 Hz), 130.2, 129.5, 129.0, 126.4, 120.2 (q, *J* = 269.6 Hz), 103.7, 43.5, 37.9, 21.4; ESI-MS calculated for C_14_H_12_ClF_3_N_3_O [M-H]^-^, 330.0626; found 330.0618.

*N-(4-chloro-1-methyl-3-(trifluoromethyl)-1H-pyrazol-5-yl)-2-(p-tolyl)acetamide* (**C-48**)

Yellow solid, yield 76%; mp = 147.2**–**150.3 ℃; ^1^H NMR (400 MHz, DMSO-*d*_6_) δ 10.48 (s, 1H), 7.24 (d, *J* = 7.8 Hz, 2H), 7.15 (d, *J* = 7.9 Hz, 2H), 3.70 (s, 2H), 3.67 (s, 3H), 2.29 (s, 3H); ^13^C NMR (101 MHz, DMSO-*d*_6_) δ 171.1, 136.3, 136.2, 135.4 (q, *J* = 37.1 Hz), 132.4, 129.5, 120.9 (q, *J* = 268.8 Hz), 103.2, 41.8, 37.7, 21.1; ESI-MS calculated for C_14_H_12_ClF_3_N_3_O [M-H]^-^, 330.0626; found 330.0617.

*N-(4-fluoro-1-methyl-3-(trifluoromethyl)-1H-pyrazol-5-yl)-2-(m-tolyl)acetamide* (**C-49**)

White solid, yield 85 %; mp = 101.0**–**103.5 ℃; ^1^H NMR (400 MHz, Chloroform-*d*) δ 7.30 (t, *J* = 7.5 Hz, 1H), 7.15 (t, *J* = 12.8 Hz, 3H), 6.93 (s, 1H), 3.74 (s, 2H), 3.64 (s, 3H), 2.38 (s, 3H); ^13^C NMR (101 MHz, DMSO-*d*_6_) δ 166.2, 134.7, 134.4 (d, *J* = 257.1 Hz), 128.5, 125.3, 124.6, 124.2, 122.3 (qd, *J* = 39.9, 6.0 Hz), 121.5, 117.7 (q, *J* = 22.2 Hz), 38.5, 32.6, 16.6; ESI-MS calculated for C_14_H_12_F_4_N_3_O [M-H]^-^, 314.0922; found 314.0913.

*N-(4-fluoro-1-methyl-3-(trifluoromethyl)-1H-pyrazol-5-yl)-2-(p-tolyl)acetamide* (**C-50**)

Yellow solid, yield 77%; mp = 126.2**–**128.3 ℃; ^1^H NMR (400 MHz, DMSO-*d*_6_) δ 10.34 (s, 1H), 7.22 (d, *J* = 7.8 Hz, 2H), 7.15 (d, *J* = 7.9 Hz, 2H), 3.68 (s, 2H), 3.65 (s, 3H), 2.28 (s, 3H); ^13^C NMR (101 MHz, DMSO-*d*_6_) δ 171.0, 138.6 (d, *J* = 250.9 Hz), 136.4, 132.3, 129.5, 129.4, 126.0, 125.9, 125.4 (qd, *J* = 38.6, 6.1 Hz), 124.9 (d, *J* = 22.3 Hz), 120.7 (qd, *J* = 267.8, 3.5 Hz), 41.8, 37.6, 21.1; ESI-MS calculated for C_14_H_12_F_4_N_3_O [M-H]^-^, 314.0922; found 314.0913.

Figure S1. ^1^H NMR of compound **C-1** in DMSO-*d*_6_

Figure S2. ^13^C NMR of compound **C-1** in DMSO-*d*_6_

Figure S3. HRMS (ESI) for compound **C-1**

Figure S4. ^1^H NMR of compound **C-2** in DMSO-*d*_6_

Figure S5. ^13^C NMR of compound **C-2** in DMSO-*d*_6_

Figure S6. HRMS (ESI) for compound **C-2**

Figure S7. ^1^H NMR of compound **C-3** in DMSO-*d*_6_

Figure S8. ^13^C NMR of compound **C-3** in DMSO-*d*_6_

Figure S9. HRMS (ESI) for compound **C-3**

Figure S10. ^1^H NMR of compound **C-4** in DMSO-*d*_6_

Figure S11. ^13^C NMR of compound **C-4** in DMSO-*d*_6_

Figure S12. HRMS (ESI) for compound **C-4**

Figure S13. ^1^H NMR of compound **C-5** in DMSO-*d*_6_

Figure S14. ^13^C NMR of compound **C-5** in DMSO-*d*_6_

Figure S15. HRMS (ESI) for compound **C-5**

Figure S16. ^1^H NMR of compound **C-6** in DMSO-*d*_6_

Figure S17. ^13^C NMR of compound **C-6** in DMSO-*d*_6_

Figure S18. HRMS (ESI) for compound **C-6**

Figure S19. ^1^H NMR of compound **C-7** in DMSO-*d*_6_

Figure S20. ^13^C NMR of compound **C-7** in DMSO-*d*_6_

Figure S21. HRMS (ESI) for compound **C-7**

Figure S22. ^1^H NMR of compound **C-8** in DMSO-*d*_6_

Figure S23. ^13^C NMR of compound **C-8** in DMSO-*d*_6_

Figure S24. HRMS (ESI) for compound **C-8**

Figure S25. ^1^H NMR of compound **C-9** in DMSO-*d*_6_

Figure S26. ^13^C NMR of compound **C-9** in DMSO-*d*_6_

Figure S27. HRMS (ESI) for compound **C-9**

Figure S28. ^1^H NMR of compound **C-10** in DMSO-*d*_6_

Figure S29. ^13^C NMR of compound **C-10** in DMSO-*d*_6_

Figure S30. HRMS (ESI) for compound **C-10**

Figure S31. ^1^H NMR of compound **C-11** in DMSO-*d*_6_

Figure S32. ^13^C NMR of compound **C-11** in DMSO-*d*_6_

Figure S33. HRMS (ESI) for compound **C-11**

Figure S34. ^1^H NMR of compound **C-12** in DMSO-*d*_6_

Figure S35. ^13^C NMR of compound **C-12** in DMSO-

Figure S36. HRMS (ESI) for compound **C-12**

Figure S37. ^1^H NMR of compound **C-13** in DMSO-*d*_6_

Figure S38. ^13^C NMR of compound **C-13** in DMSO-*d*

Figure S39. HRMS (ESI) for compound **C-13**

Figure S40. ^1^H NMR of compound **C-14** in DMSO-*d*_6_

Figure S41. ^13^C NMR of compound **C-14** in DMSO-*d*_6_

Figure S42. HRMS (ESI) for compound **C-14**

Figure S43. ^1^H NMR of compound **C-15** in DMSO-*d*_6_

Figure S44. ^13^C NMR of compound **C-15** in DMSO-*d*_6_

Figure S45. HRMS (ESI) for compound **C-15**

Figure S46. ^1^H NMR of compound **C-16** in DMSO-*d*_6_

Figure S47. ^13^C NMR of compound **C-16** in DMSO-*d*_6_

Figure S48. HRMS (ESI) for compound **C-16**

Figure S49. ^1^H NMR of compound **C-17** in DMSO-*d*_6_

Figure S50. ^13^C NMR of compound **C-17** in DMSO-*d*_6_

Figure S51. HRMS (ESI) for compound **C-17**

Figure S52. ^1^H NMR of compound **C-18** in DMSO-*d*_6_

Figure S53. ^13^C NMR of compound **C-18** in DMSO-*d*_6_

Figure S54. HRMS (ESI) for compound **C-18**

Figure S55. ^1^H NMR of compound **C-19** in DMSO-*d*_6_

Figure S56. ^13^C NMR of compound **C-19** in DMSO-*d*_6_

Figure S57. HRMS (ESI) for compound **C-19**

Figure S58. ^1^H NMR of compound **C-20** in DMSO-*d*_6_

Figure S59. ^13^C NMR of compound **C-20** in DMSO-*d*_6_

Figure S60. HRMS (ESI) for compound **C-20**

Figure S61. ^1^H NMR of compound **C-21** in DMSO-*d*_6_

Figure S62. ^13^C NMR of compound **C-21** in DMSO-*d*_6_

Figure S63. HRMS (ESI) for compound **C-21**

Figure S64. ^1^H NMR of compound **C-22** in DMSO-*d*_6_

Figure S65. ^13^C NMR of compound **C-22** in DMSO-*d*_6_

Figure S66. HRMS (ESI) for compound **C-22**

Figure S67. ^1^H NMR of compound **C-23** in DMSO-*d*_6_

Figure S68. ^13^C NMR of compound **C-23** in DMSO-*d*_6_

Figure S69. HRMS (ESI) for compound **C-23**

Figure S70. ^1^H NMR of compound **C-24** in DMSO-*d*_6_

Figure S71. ^13^C NMR of compound **C-24** in DMSO-*d*_6_

Figure S72. HRMS (ESI) for compound **C-24**

Figure S73. ^1^H NMR of compound **C-25** in DMSO-*d*_6_

Figure S74. ^13^C NMR of compound **C-25** in DMSO-*d*_6_

Figure S75. HRMS (ESI) for compound **C-25**

Figure S76. ^1^H NMR of compound **C-26** in DMSO-*d*_6_

Figure S77. ^13^C NMR of compound **C-26** in DMSO-*d*_6_

Figure S78. HRMS (ESI) for compound **C-26**

Figure S79. ^1^H NMR of compound **C-27** in DMSO-*d*_6_

Figure S80. ^13^C NMR of compound **C-27** in DMSO-*d*_6_

Figure S81. HRMS (ESI) for compound **C-27**

Figure S82. ^1^H NMR of compound **C-28** in DMSO-*d*_6_

Figure S83. ^13^C NMR of compound **C-28** in DMSO-*d*_6_

Figure S84. HRMS (ESI) for compound **C-28**

Figure S85. ^1^H NMR of compound **C-29** in DMSO-*d*_6_

Figure S86. ^13^C NMR of compound **C-29** in DMSO-*d*_6_

Figure S87. HRMS (ESI) for compound **C-29**

Figure S88. ^1^H NMR of compound **C-30** in DMSO-*d*_6_

Figure S89. ^13^C NMR of compound **C-30** in DMSO-*d*_6_

Figure S90. HRMS (ESI) for compound **C-30**

Figure S91. ^1^H NMR of compound **C-31** in DMSO-*d*_6_

Figure S92. ^13^C NMR of compound **C-31** in DMSO-*d*_6_

Figure S93. HRMS (ESI) for compound **C-31**

Figure S94. ^1^H NMR of compound **C-32** in DMSO-*d*_6_

Figure S95. ^13^C NMR of compound **C-32** in DMSO-*d*_6_

Figure S96. HRMS (ESI) for compound **C-32**

Figure S97. ^1^H NMR of compound **C-33** in DMSO-*d*_6_

Figure S98. ^13^C NMR of compound **C-33** in DMSO-*d*_6_

Figure S99. HRMS (ESI) for compound **C-33**

Figure S100. ^1^H NMR of compound **C-34** in DMSO-*d*_6_

Figure S101. ^13^C NMR of compound **C-34** in DMSO-*d*_6_

Figure S102. HRMS (ESI) for compound **C-34**

Figure S103. ^1^H NMR of compound **C-35** in DMSO-*d*_6_

Figure S104. ^13^C NMR of compound **C-35** in DMSO-*d*_6_

Figure S105. HRMS (ESI) for compound **C-35**

Figure S106. ^1^H NMR of compound **C-36** in DMSO-*d*_6_

Figure S107. ^13^C NMR of compound **C-36** in DMSO-*d*_6_

Figure S108. HRMS (ESI) for compound **C-36**

Figure S109. ^1^H NMR of compound **C-37** in DMSO-*d*_6_

Figure S110. ^13^C NMR of compound **C-37** in DMSO-*d*_6_

Figure S111. HRMS (ESI) for compound **C-37**

Figure S112. ^1^H NMR of compound **C-38** in DMSO-*d*_6_

Figure S113. ^13^C NMR of compound **C-38** in DMSO-*d*_6_

Figure S114. HRMS (ESI) for compound **C-38**

Figure S115. ^1^H NMR of compound **C-39** in DMSO-*d*_6_

Figure S116. ^13^C NMR of compound **C-39** in DMSO-*d*_6_

Figure S117. HRMS (ESI) for compound **C-39**

Figure S118. ^1^H NMR of compound **C-40** in DMSO-*d*_6_

Figure S119. ^13^C NMR of compound **C-40** in DMSO-*d*_6_

Figure S120. HRMS (ESI) for compound **C-40**

Figure S121. ^1^H NMR of compound **C-41** in DMSO-*d*_6_

Figure S122. ^13^C NMR of compound **C-41** in DMSO-*d*_6_

Figure S123. HRMS (ESI) for compound **C-41**

Figure S124. ^1^H NMR of compound **C-42** in DMSO-*d*_6_

Figure S125. ^13^C NMR of compound **C-42** in DMSO-*d*_6_

Figure S126. HRMS (ESI) for compound **C-42**

Figure S127. ^1^H NMR of compound **C-43** in DMSO-*d*_6_

Figure S128. ^13^C NMR of compound **C-43** in DMSO-*d*_6_

Figure S129. HRMS (ESI) for compound **C-43**

Figure S130. ^1^H NMR of compound **C-44** in DMSO-*d*_6_

Figure S131. ^13^C NMR of compound **C-44** in DMSO-*d*_6_

Figure S132. HRMS (ESI) for compound **44**

Figure S133. ^1^H NMR of compound **C-45** in DMSO-*d*_6_

Figure S134. ^13^C NMR of compound **C-45** in DMSO-*d*_6_

Figure S135. HRMS (ESI) for compound **C-45**

Figure S136. ^1^H NMR of compound **C-46** in DMSO-*d*_6_

Figure S137. ^13^C NMR of compound **C-46** in DMSO-*d*_6_

Figure S138. HRMS (ESI) for compound **C-46**

Figure S139. ^1^H NMR of compound **C-47** in DMSO-*d*_6_

Figure S140. ^13^C NMR of compound **C-47** in DMSO-*d*_6_

Figure S141. HRMS (ESI) for compound **C-47**

Figure S142. ^1^H NMR of compound **C-48** in DMSO-*d*_6_

Figure S143. ^13^C NMR of compound **C-48** in DMSO-*d*_6_

Figure S144. HRMS (ESI) for compound **C-48**

Figure S145. ^1^H NMR of compound **C-49** in DMSO-*d*_6_

Figure S146. ^13^C NMR of compound **C-49** in DMSO-*d*_6_

Figure S147. HRMS (ESI) for compound **C-49**

Figure S148. ^1^H NMR of compound **C-44** in DMSO-*d*_6_

Figure S149. ^13^C NMR of compound **C-44** in DMSO-*d*_6_

Figure S150. HRMS (ESI) for compound **C-50**
